# Single-Cell Transcriptomics Reveals Peripheral Immune Responses in Anti-Synthetase Syndrome-Associated Interstitial Lung Disease

**DOI:** 10.3389/fimmu.2022.804034

**Published:** 2022-02-17

**Authors:** Lili Zhu, Zhong Cao, Shiyao Wang, Changshui Zhang, Lei Fang, Yanhong Ren, Bingbing Xie, Jing Geng, Sheng Xie, Ling Zhao, Li Ma, Huaping Dai, Chen Wang

**Affiliations:** ^1^ Department of Pulmonary and Critical Care Medicine, China-Japan Friendship Hospital, Capital Medical University, Beijing, China; ^2^ National Center for Respiratory Medicine, Beijing, China; ^3^ National Clinical Research Center for Respiratory Diseases, Institute of Respiratory Medicine, Chinese Academy of Medical Sciences, Peking Union Medical College, Beijing, China; ^4^ Institute for Artificial Intelligence, Tsinghua University (THUAI), State Key Lab of Intelligent Technologies and Systems, Beijing National Research Center for Information Science and Technology (BNRist), Beijing, China; ^5^ DataCanvas Technology Co., Ltd, Beijing, China; ^6^ Department of Radiology, China-Japan Friendship Hospital, Beijing, China; ^7^ Department of Pathology, China-Japan Friendship Hospital, Beijing, China; ^8^ Department of Rheumatology, China-Japan Friendship Hospital, Beijing, China

**Keywords:** interstitial lung disease, anti-synthetase syndrome, scRNA-seq, immune responses, differentiation of CD4 T cells

## Abstract

**Objective:**

Interstitial lung diseases (ILDs) secondary to anti-synthetase syndrome (ASS) greatly influence the prognoses of patients with ASS. Here we aimed to investigate the peripheral immune responses to understand the pathogenesis of this condition.

**Methods:**

We performed single-cell RNA sequencing (scRNA-seq) of peripheral blood mononuclear cells (PBMCs) from 5 patients with ASS-ILD and 3 healthy donors (HDs). Flow cytometry of PBMCs was performed to replenish the results of scRNA-seq.

**Results:**

We used scRNA-seq to depict a high-resolution visualization of cellular landscape in PBMCs from patients with ASS-ILD. Patients showed upregulated interferon responses among NK cells, monocytes, T cells, and B cells. And the ratio of effector memory CD8 T cells to naïve CD8 T cells was significantly higher in patients than that in HDs. Additionally, Th1, Th2, and Th17 cell differentiation signaling pathways were enriched in T cells. Flow cytometry analyses showed increased proportions of Th17 cells and Th2 cells, and decreased proportion of Th1 cells in patients with ASS-ILD when compared with HDs, evaluated by the expression patterns of chemokine receptors.

**Conclusions:**

The scRNA-seq data analyses reveal that ASS-ILD is characterized by upregulated interferon responses, altered CD8 T cell homeostasis, and involvement of differentiation signaling pathways of CD4 T cells. The flow cytometry analyses show that the proportions of Th17 cells and Th2 cells are increased and the proportion of Th1 cells is decreased in patients with ASS-ILD. These findings may provide foundations of novel therapeutic targets for patients with this condition.

## Introduction

Interstitial lung diseases (ILDs), which encompass a large spectrum of distinct etiologies, can be idiopathic or manifest as a pulmonary complication of a connective tissue disease (CTD), such as idiopathic inflammatory myositis (IIM) (1, 2). With the discovery of myositis specific antibodies (MSAs) and development of more commercially available assays for MSAs, anti-synthetase syndrome (ASS) has been recognized as an important cause of ILDs (3). ASS is characterized by myositis, ILD, “mechanic’s hands”, fever, arthritis, and Raynaud’s phenomenon (4). The hallmark in the diagnosis of ASS is detecting an anti-synthetase antibody in the serum of patients. Up to now, there are 8 anti-synthetase antibodies, including anti-histidyl (Jo-1), anti-threonyl (PL-7), anti-alanyl (PL-12), anti-isoleucyl (OJ), anti-glycyl (EJ), anti-asparaginyl (KS), anti-phenylalanyl (Zo), and anti-tyrosyl (Ha)-tRNA-synthetase antibody (5). ILD is an increasingly recognized complication of ASS, and it is reported that 67-100% of ASS patients develop ILD (6). Besides, ILD is the leading cause of deaths related to ASS (7). The most frequent high-resolution computed tomography (HRCT) pattern of ASS-associated ILD (ASS-ILD) is non-specific interstitial pneumonia (NSIP), followed by organizing pneumonia (OP); usual interstitial pneumonia (UIP), and concomitant NSIP and OP are less common HRCT patterns (8). ILD may occur alone without signs of muscle or skin involvement, to differentiate ASS-ILD from other types of ILD is not easy but of great significance (9). Early diagnosis, timely intervention, regular screening, and referral to promising clinical trials might help to improve the prognoses of patients with ASS-ILD.

Although great advances have been made in the pathogenesis of this condition, there remain substantial gaps in our knowledge of ASS-ILD. A prospective Pittsburgh ASS cohort study reported that 33% (66/202) of patients died and 6% (12/202) of patients received lung transplantation from 1985 to 2009. The 5-year and 10-year unadjusted cumulative survival rate was 84% and 61%, respectively. ILD is associated with high mortality, 49% of mortality was attributed to pulmonary fibrosis in the cohort study (7). The mainstay of treatment regimens for ASS is glucocorticoids combined with immunosuppressant or biological agents, including azathioprine, calcineurin inhibitors, methotrexate, cyclophosphamide, mycophenolate mofetil, and rituximab (10, 11). Options for ASS-ILD patients who are refractory to conventional therapies are lacking, further investigations into the pathogenesis of this condition may help to target specific pathogenic pathways.

Dysregulated immune equilibriums contribute to the development of CTDs (12). The pathogenic roles of autoantigen and auto-reactive CD4 T cells in ASS have been proposed (13, 14). However, a global visualization of the pathogenic immune responses has not been reported. Here, we used single-cell RNA sequencing (scRNA-seq) technology to depict a high-resolution visualization of cellular landscape in peripheral blood mononuclear cells (PBMCs) from patients with ASS-ILD, which will facilitate a better understanding of the pathogenesis of this condition.

## Materials and Methods

### Subjects and Sample Collection

This study was approved by the ethics committee of the China-Japan Friendship Hospital, China (2019-123-K85), and each donor signed written informed consent. All patients who were hospitalized at the department of pulmonary and critical care medicine, China-Japan Friendship Hospital, China, were consecutively enrolled after the successful screening. The patients were divided into two cohorts, one is the single-cell RNA sequencing (scRNA-seq) cohort, and the other is the flow cytometry cohort. In our scRNA-seq cohort, five patients with anti-synthetase syndrome-associated interstitial lung disease (ASS-ILD) and three healthy donors (HDs) were recruited. There are overlapping chest radiological manifestations between ASS-ILD and idiopathic interstitial pneumonia (IIP), and clues for differential diagnoses are of great significance. So we enrolled patients with IIP as disease controls in the flow cytometry cohort. In our flow cytometry cohort, sixteen patients with ASS-ILD, ten patients with idiopathic non-specific interstitial pneumonia (iNSIP), ten patients with idiopathic pulmonary fibrosis (IPF), five patients with cryptogenic organizing pneumonia (COP), and ten HDs were recruited. To be included in this study, ASS-ILD and IIP (including iNSIP, COP, and IPF) must be diagnosed by a multi-disciplinary team (MDT), including at least two pulmonologists, a radiologist, a pathologist, and a rheumatologist. ASS was defined according to Connors GR’s diagnostic criteria, which included strong positive testing for an anti-synthetase antibody (identified by immunoblotting assays) and one or more of the following conditions: myositis, ILD, arthritis, unexplained and persistent fever, Raynaud phenomenon, and “Mechanic’s hands” (15). In both cohorts, all patients received no immunosuppressive therapies prior to our enrollment. And patients vaccinated within 3 months, with malignancy, infection, or other autoimmune diseases were all excluded. Chest high-resolution computed tomography (HRCT) pattern was categorized as usual interstitial pneumonia (UIP), NSIP, organizing pneumonia (OP), and NSIP/OP pattern according to the 2013 American Thoracic Society (ATS) classification of IIP independently by at least a radiologist and two pulmonologists. The classification of different entities of IIP was also determined according to ATS classification criteria (16, 17). In the scRNA-seq cohort, 10 ml of peripheral venous blood was collected from each sample, and peripheral blood mononuclear cells (PBMCs) were immediately isolated using density-gradient centrifugation with Ficoll-Hypaque according to the manufacturer’s protocol. For each sample, cell viability exceeded 90%. In the flow cytometry cohort, 6 ml of peripheral venous blood was collected from each sample, and PBMCs were isolated using density-gradient centrifugation with Ficoll-Paque Plus (GE Healthcare) according to the manufacturer’s protocol; then cells were frozen in fetal bovine serum (FBS) with 10% dimethyl sulfoxide (DMSO) for storage in liquid nitrogen until use.

### Single-Cell RNA Sequencing

Using a Chromium Next GEM Single Cell 5’ Library and Gel Bead Kit v1.1 (10X Genomics, 1000165) and Chromium Next GEM Chip G Single Cell Kit (10X Genomics, 1000127), we loaded the fresh cell suspension (300 to 600 living cells per microliter counted by Count Star) onto a Chromium single cell controller (10X Genomics) to generate single-cell gel beads in the emulsion (GEMs) in accordance with the manufacturer’s protocol. Briefly, we suspended single cells in phosphate-buffered saline (PBS) containing 0.04% bovine serum albumin (BSA). And about 10,000 cells were added to the individual channel and approximately 5,000 target cells were recovered. Captured cells were then lysed and the released RNA was barcoded through the process of reverse transcription (RT) in each GEM. RT was conducted on a S1000TM Touch Thermal Cycler (Bio Rad) at 53°C for 45 min, followed by 85°C for 5 min and held at 4°C. The complementary DNA (cDNA) was generated and amplified, after that, quality assessment was performed using an Agilent 4200 (performed by CapitalBio Technology, Beijing). According to the manufacturer’s introduction, scRNA-seq libraries were constructed using a Single Cell 5′ Library and Gel Bead kit. The libraries were then sequenced using an Illumina Novaseq6000 sequencer with a sequencing depth of at least 100,000 reads per cell with a pair-end 150-bp (PE150) reading strategy (performed by CapitalBio Technology, Beijing).

### Single-Cell RNA Sequencing Transcriptome Analysis

Raw gene expression matrices of each sample were aggregated using Cell Ranger (v.6.0.0) Pipeline coupled with human reference version GRCh38. The merged matrices were transferred to the R statistical environment for further analyses using the Seurat package (v.4.0.3). And genes expressed at more than 0.1% of the cells and cells with more than 200 genes detected were selected for further analyses. Cells with >10% mitochondrial unique molecular identifiers (UMIs), >12000 UMIs, or over 3000 or below 500 genes were removed as low-quality cells (**Supplementary Figure 1A**). After removal of low-quality cells, gene expression metrices were normalized using the SCTtransform normalization, and 3,000 highly variable genes (HVGs) were identified by the FindVariableFeatures function. Batch effects were corrected by SCT Integration (**Supplementary Figure 1B**). In order to reduce the dimension of the datasets, principal component analysis (PCA) was performed with HVGs by the ScaleData function. Cells were then clustered using the FindNeighbors and FindClusters functions with a resolution of 0.4. After that, clusters were projected into a two-dimensional plot for visualization using the RunUMAP function.

### Cell-Type Annotation and Cluster Marker Identification

Cells were clustered using the FindNeighbors and FindClusters functions in Seurat, and non-linear dimensional reduction was conducted with the RunUMAP function. Cells were clustered together according to common transcriptome features, and FindAllMarkers function was performed to find marker genes for each cluster. Clusters were then identified and annotated according to the expression of canonical markers. Cells expressing more than one canonical cell-type markers were regarded as doublets and excluded for further analysis. Naïve T cells, central memory T cells, effector memory T cells, *γδ* T cells, and proliferative T cells were combined. After integration, PCA and sub-clustering were performed as described above. PCA and sub-clustering were also conducted in B cells.

### DEG Identification and Functional Enrichment

Differentially expressed genes (DEGs) were analyzed using the FindMarkers function with parameter ‘test. use = Wilcox’ by default. The false discovery rate (FDR) adjusted p-value was calculated by the Bonferroni correction method. DEGs were calculated using a minimum log (fold change) of 0.3 and a maximum FDR adjusted p-value of 0.05. Then the upregulated expressed genes were mapped to terms in the Geno Ontology (GO) database. Only GO Biological Process was used in this study. Kyoto Encyclopedia of Genes and Genomes (KEGG) pathway analysis was performed in the same way as GO.

### Antibodies and Flow Cytometry Analysis

Cells recovered from liquid nitrogen were incubated with human Fc Block (BD Pharmingen) after being washed by Dulbecco’s PBS (D-PBS). BD Horizon™ Fixable Viability Stain 510 (FVS510) was used for the exclusion of dead cells. Each sample was then stained for expressions of cell surface markers, including CD3 (BUV 395), CD4 (BUV496), CD45RA (BV711), CCR6 (BB515), CCR4 (BV421), CXCR3 (PE), and intracellular marker of Foxp3 (Alexa Fluor 647). Prior to intracellular staining, Transcription Factor Buffer Set (BD Pharmingen) was used for cell fixing and permeabilization. Fluorescence minus one (FMO) controls for CD3, CD4, CD45RA, CCR6, CCR4, CXCR3, and Foxp3 were used. Single-stained Comp Beads (Anti-Mouse Ig, κ/Negative Control Compensation Particles Set, BD) were applied for compensation controls. Each T cell subset was defined as follows: CD3+CD4+ T cells were selected according to CD3 and CD4 expressions in lymphocytes. Five CD3+CD4+ T cell subsets were defined according to CD45RA and Foxp3 expressions, including a naïve T cell subset (CD45RA^+^Foxp3^-^), a memory T cell subset (CD45RA^-^Foxp3^-^), an activated T cell subset (CD45RA^-^Foxp3^int^), a naïve Treg cell subset (CD45RA^+^Foxp3^int^), and an activated Treg cell subset (CD45RA^-^Foxp3^high^). The total memory CD4 T cells included the memory T cell subset and activated T cell subset. Distinct Th cell subsets of total memory T cells were defined according to chemokine-receptor expressions. CCR6- total memory T cells were divided into two subsets, including a Th1 cell subset (CXCR3+CCR4-), and a Th2 cell subset (CXCR3-CCR4+). CCR6+ total memory T cells were divided into three subsets, including a Th17 subset (CXCR3-CCR4+), a Th17.1 cell subset (CXCR3+CCR4-), and a double positive cell subset (CXCR3+CCR4+) (18). Monoclonal antibodies were purchased from BD Pharmingen, and we processed PBMCs in accordance with the manufacturer’s protocol. Cells were analyzed using FlowJo software version 10.8 or Becton Dickenson FACS DIVA software version 7.0.

### Statistical Analysis

We used the Mann-Whitney test to compare differences between ASS-ILD group and different control groups (iNSIP group, COP group, IPF group, and HD group) for continuous variables, respectively. Analyses were determined using GraphPad Prism version 9. The results were regarded as statistically significant when a two-sided p-value was <0.05.

## Results

### Single-Cell Transcriptomic Profiles of PBMCs

**Figure 1:**
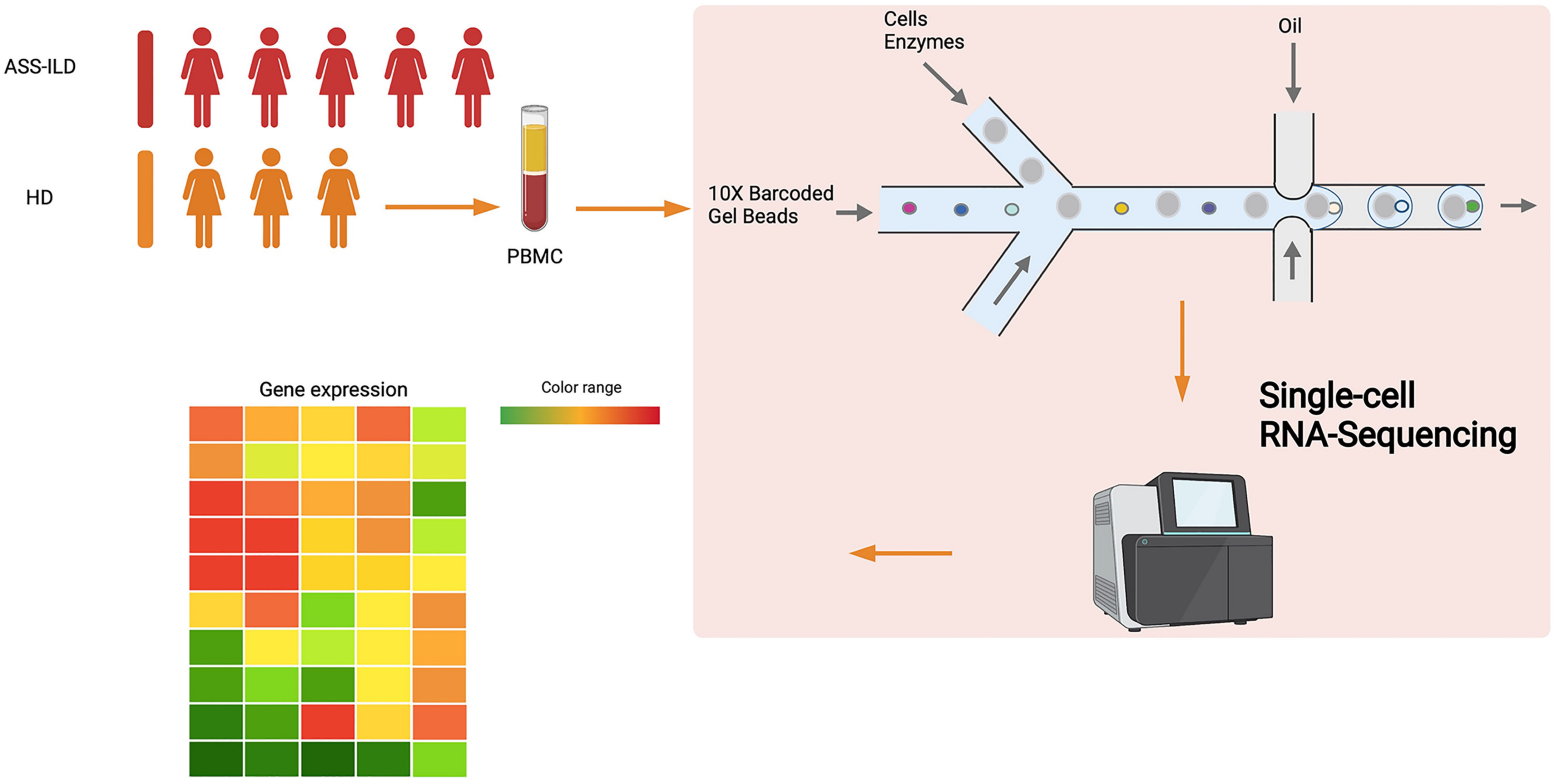
Schematic of overall experimental workflow.

**Figure 2:**
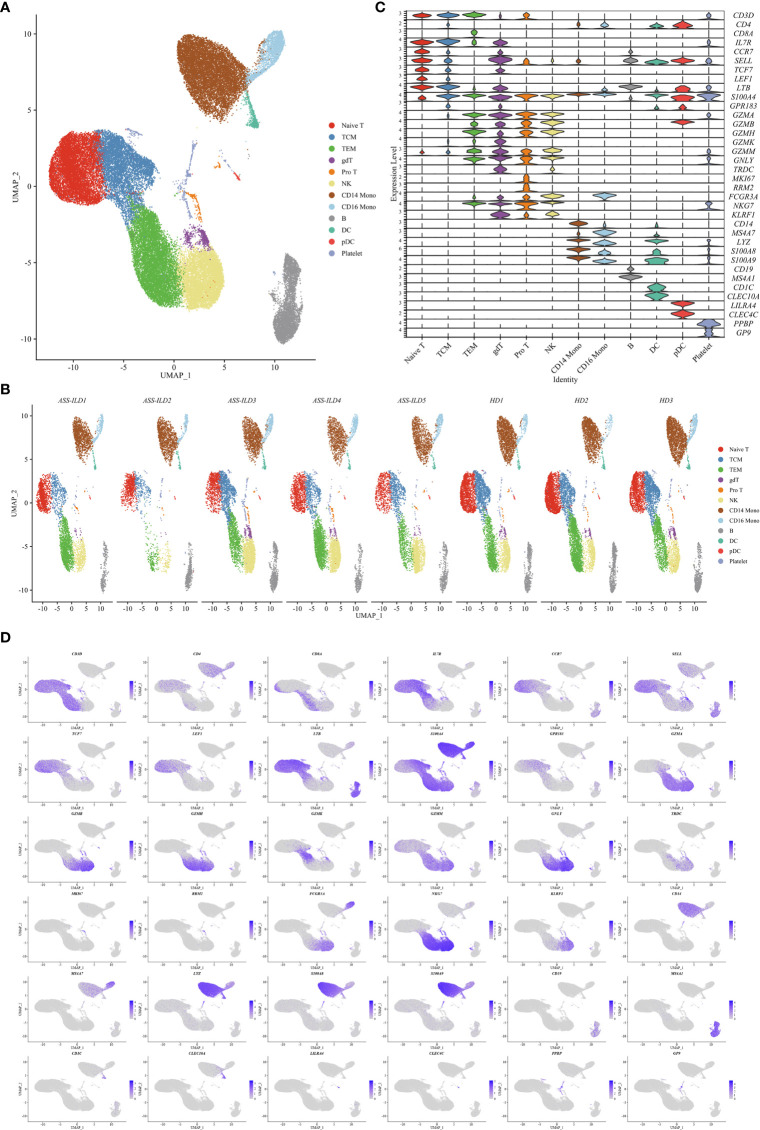
Single-cell RNA sequencing of PBMCs from patients with ASS-ILD and HDs. **(A)** A two-dimensional UMAP plot for visualization of 52973 cells from patients with ASS-ILD (n=5) and HDs (n=3). **(B)** Annotating condition of each patient with ASS-ILD and HD. **(C)** Violin plot depicting the expression distribution of selected marker genes in the 12 clusters. The rows represent selected marker genes and the columns represent clusters with the same color as in **(A)**. **(D)** Expressions of canonical marker genes for 12 major cell types as represented in the UMAP plot. Data are colored according to expression levels and the legend is labeled in log scale. PBMC, peripheral blood mononuclear cell; ASS-ILD, anti-synthetase syndrome-associated interstitial lung disease; HD, healthy donor; UMAP, uniform manifold approximation and projection; TCM, central memory T lymphocyte; TEM, effector memory T lymphocyte; pro T, proliferative T cell; NK, natural killer cell; mono, monocyte; DC, dendritic cell; pDC, plasmacytoid DC.

To characterize the immunological features of patients with ASS-ILD, we performed scRNA-seq (10X Genomics) on PBMCs from 5 patients with ASS-ILD (ASS-ILD 1-5) and 3 healthy donors (HD 1-3) (**Figure 1** and **Supplementary Figure 2A**). The clinical manifestations, laboratory features, chest HRCT patterns, and pulmonary function test (PFT) parameters of recruited patients and HDs were detailed in **Supplementary Tables 1** and **2**. We analyzed a whole of 52973 PBMCs from 5 ASS-ILD and 3 HD samples. Poor-quality cells were filtered out, we integrated all high-quality cells into an unbiased and comparable dataset. Using uniform manifold approximation and projection (UMAP), we identified the transcriptomes of 14 clusters, and 2 of them were identified as doublets (**Supplementary Figures 2B, C**). Thus 12 major cell types were clustered and annotated based on the expressions of canonical marker genes (**Figures 2A, B** **)**. These cells included naïve T lymphocytes (Naïve T, CD3D+CCR7+), central memory T lymphocytes (TCM, CD3D+CCR7+S100A4+), effector memory T lymphocytes (TEM, CD3D+ S100A4+ GZMA+), γδ T cells (gdT, TRDC+), proliferative T cells (pro T, CD3D+MKI67+), natural killer cells (NK, KLRF1+FCGR3A+), CD14+ monocytes (CD14 mono, LYZ+CD14+), CD16+ monocytes (CD16 mono, LYZ+FCGR3A+), B cells (CD19+MS4A1+), dendritic cells (DC, CD1C+CLEC10A+), plasmacytoid DCs (pDC; LILRA4+CLEC4C+), and platelets (PPBP+GP9+) (**Figures 2C, D** **)**. In downstream analyses, platelets were excluded. To reveal cell compositions between patients with ASS-ILD and HDs, we compared the relative percentages of the 12 cell types between them. No significant differences in cell compositions were found (**Supplementary Figures 3A, B**).

### Features of NK Cells and Monocytes in Patients With ASS-ILD

**Figure 3:**
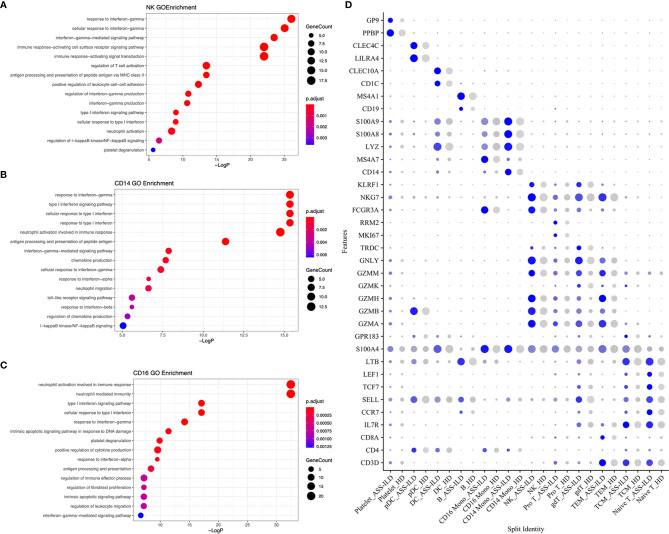
GO enrichment analyses of the upregulated genes in patients with ASS-ILD. **(A)** GO enrichment analysis for NK cells. **(B)** GO enrichment analysis for CD14 monocytes. **(C)** GO enrichment analysis for CD16 monocytes. **(D)** Dot plot depicting average expression and percentage of expressed cells of selected marker genes in each labeled cell subset. GO, Geno Ontology; ASS-ILD, anti-synthetase syndrome-associated interstitial lung disease; NK, natural killer.

Among innate immune cells, CD14 mono, CD16 mono, and NK cells were the most three prevalent subsets, and next, we further characterized the transcriptomic features of these three subsets. We first compared the expression patterns of the patients with those of the HDs in NK cells. We found that significantly differentially expressed genes (DEGs) were involved in responses to type I and II interferons, neutrophil activation, and antigen processing and presentation of peptide antigen *via* MHC class II in patients with ASS-ILD using Geno Ontology (GO) analysis (**Figure 3A**). For CD14+ and CD16+ monocytes, DEGs were associated with responses to type I and II interferons, neutrophil activation, and chemokine or cytokine production (**Figures 3B, C** **)**. A dot plot showed the average expression and percentage of expressed cells of selected marker genes in each labeled cell subset (**Figure 3D**). IFN-signature appears a typical feature of IIM, and it varies among different subtypes (19). In our study, we found that monocytes and NK cells both showed upregulated responses to type I and II interferons. These results suggest that innate immune cell types in patients with ASS-ILD show consistent inflammatory responses, like upregulated responses to interferons.

### Features of T Cell Subsets in Patients With ASS-ILD

**Figure 4:**
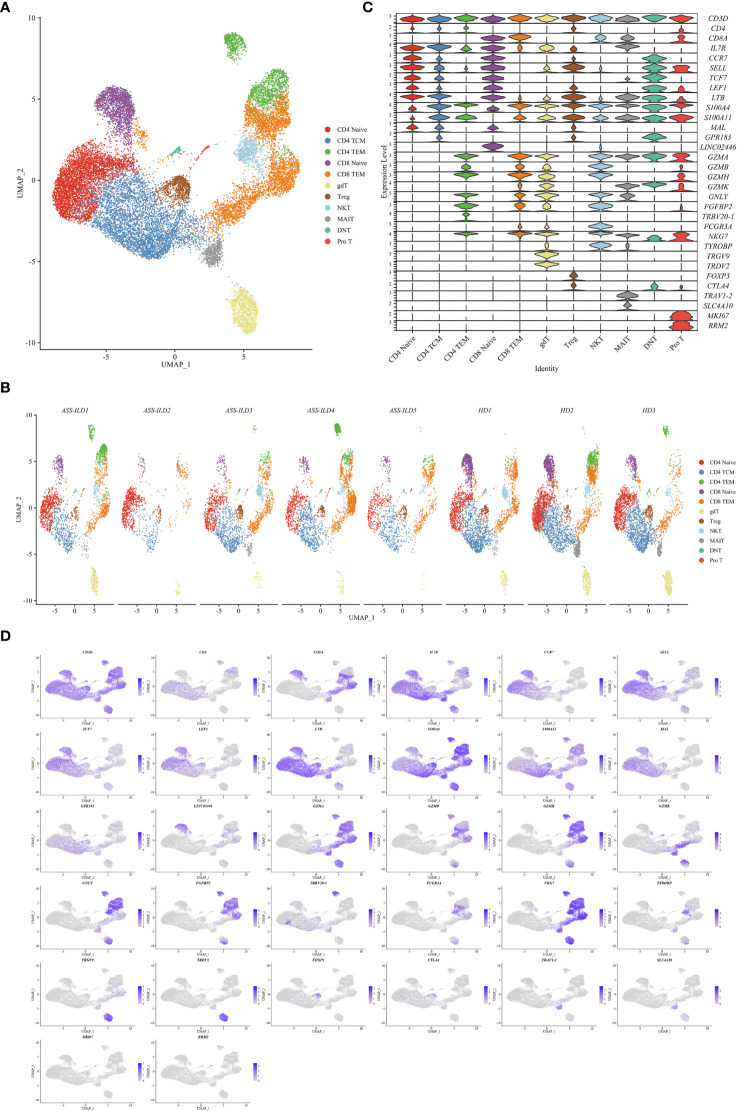
Immunological features of T cell subsets. **(A)** UMAP visualization of 26843 T cells, including 4 CD4+ T cell subsets, 2 CD8+ T cell subsets, and 5 other T cell subsets. **(B)** Annotating condition of each patient with ASS-ILD and HD. **(C)** Violin plot depicting the expression distribution of selected marker genes in the 11 T cell subsets. The rows represent selected marker genes and the columns represent clusters with the same color as in **(A)**. **(D)** Expression of canonical marker genes for 11 T cell subsets as represented in the UMAP plot. Data are colored according to expression levels and the legend is labeled in log scale. UMAP, uniform manifold approximation and projection; ASS-ILD, anti-synthetase syndrome-associated interstitial lung disease; HD, healthy donor; TCM, central memory T lymphocyte; TEM, effector memory T lymphocyte; Treg, regulatory CD4 T lymphocyte; NKT, natural killer T cell; MAIT, mucosal-associated invariant T cell; DNT, double negative T cell; pro T, proliferative T cell.

**Figure 5:**
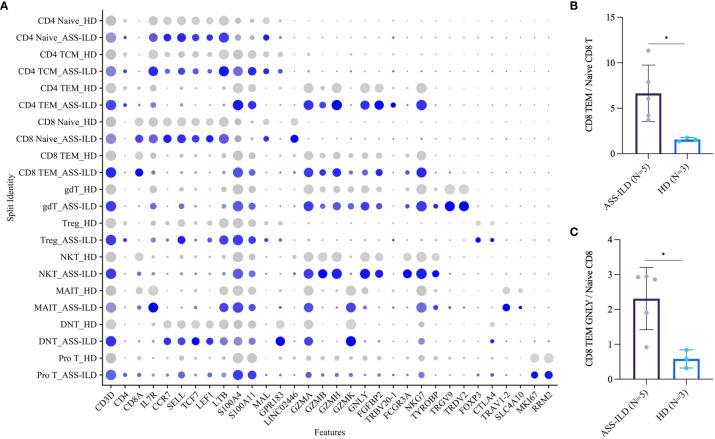
Comparisons of T cell subsets between patients with ASS-ILD and HDs. **(A)** Dot plot depicting the average expression and percentage of expressed cells of selected marker genes in each labeled T cell subset. **(B)** Box plot of the ratios of CD8 TEM cells to CD8 Naïve T cells in patients with ASS-ILD and HDs, the result calculated by the Mann-Whitney test. The result was considered significant when a two-sided p-value was <0.05. **(C)** Box plot of the ratios of CD8 TEM GNLY cells to CD8 Naïve T cells, analysis similar to **(B)** ASS-ILD, anti-synthetase syndrome-associated interstitial lung disease; HD, healthy donor; TEM, effector memory T. *p < 0.05.

To trace the transcriptomic changes of different T subtypes, we sub-clustered T cells into 18 subsets and those with similar T cell markers were categorized together (**Supplementary Figures 4A–H**). Then we identified 11 subsets of T cells based on the expressions of canonical T cell markers: 4 subsets of CD4+ T lymphocytes (CD3D+CD4+), 2 subsets of CD8+ T lymphocytes (CD3D+CD8A+), and 5 subsets of other T cells (**Figures 4A, B** **)**. The 4 subsets of CD4+ T lymphocytes included naïve CD4+ T lymphocytes (CD4 Naïve, CCR7+SELL+), and central memory CD4+ T lymphocytes (CD4 TCM, CCR7+SELL+S100A4+), effector memory CD4+ T lymphocytes (CD4 TEM, S100A4+GZMA+), and regulatory CD4 T lymphocytes (Treg, FOXP3+CTLA4+); the 2 subsets of CD8+ T lymphocytes included naïve CD8 T lymphocytes (CD8 Naïve, CCR7+SELL+), and effector memory CD8+ T lymphocytes (CD8 TEM, S100A4+GZMA+); other T cells included *γδ* T cells (TRGV9+TRDV2+), NKT cells (CD3D+NKG7+TYROBP+), mucosal-associated invariant T (MAIT) cells (TRAV1-2+SLC4A10+), double-negative T cells (DNT, CD4-CD8A-), and pro T (MKI67+RRM2+) (**Figures 4C, D** **)**. A dot plot showed the average expression and percentage of expressed cells of selected marker genes in each labeled T cell subset (**Figure 5A**). No significant differences in cell compositions were found in each cluster except pro T cells (**Supplementary Figures 5A, B**). However, the number of pro T cells was too small to get a robust result, so we didn’t perform further analysis. Next, we compared the expression of CD4 and CD8 between patients with ASS-ILD and HDs, no significant differences were found (**Supplementary Figure 5C**). Then the ratios of CD4 TCM to CD4 Naïve, CD4 TEM to CD4 Naïve, CD8 TEM to CD8 Naïve were compared between patients with ASS-ILD and HDs, respectively. Only the differences in the ratio of CD8 TEM to CD8 Naïve were statistically significant (**Figure 5B**). After that, we further compared the ratios of CD8 TEM GZMK, CD8 TEM GNLY, and CD8 TEM TRAV27 to CD8 Naïve between patients with ASS-ILD and HDs, respectively; only the differences in the ratio of CD8 TEM GNLY to CD8 Naïve were statistically significant between patients and HDs (**Figure 5C**).

**Figure 6:**
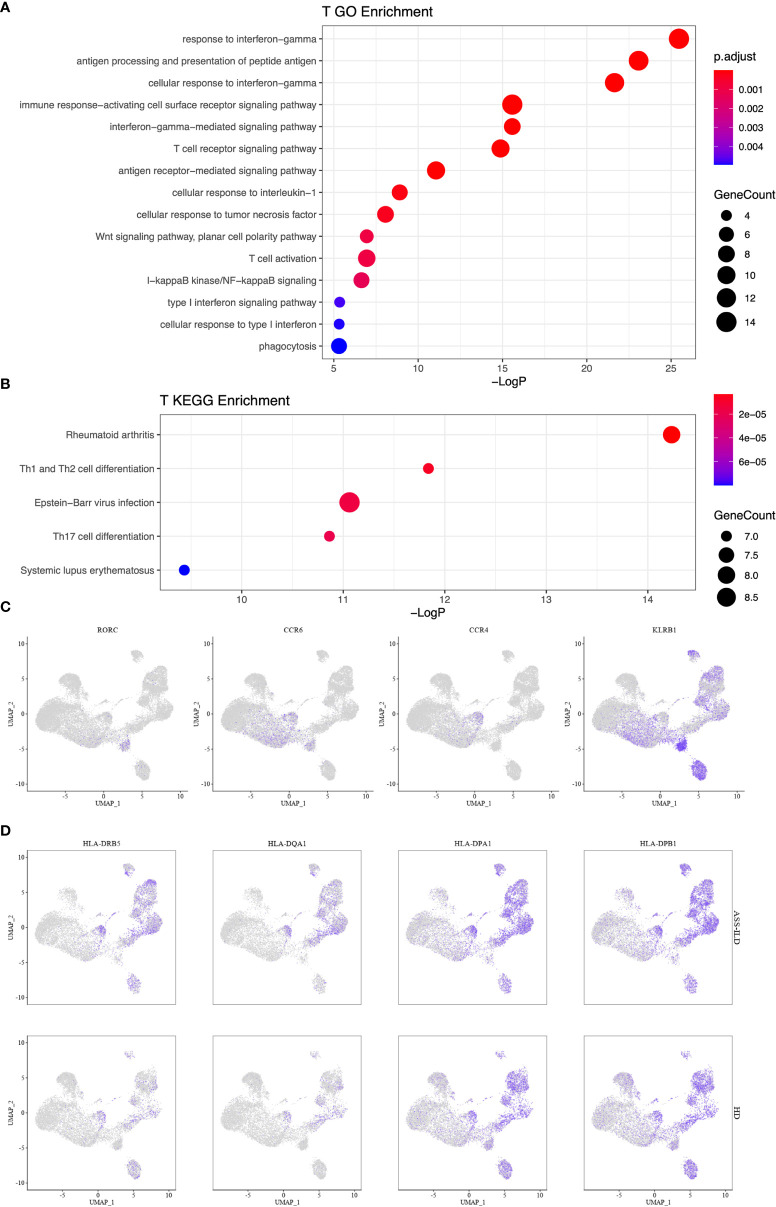
Enrichment analyses for T cells. **(A)** GO enrichment analysis of upregulated genes for T cells. **(B)** KEGG pathway enrichment analysis of upregulated genes for T cells. **(C)** Th17 cell-related gene expressions in the UMAP plot. **(D)** Enriched genes associated with Th1, Th2, and Th17 cell differentiation signaling pathways. GO, Geno Ontology; KEGG, Kyoto Encyclopedia of Genes and Genomes; UMAP, uniform manifold approximation and projection.

**Figure 7:**
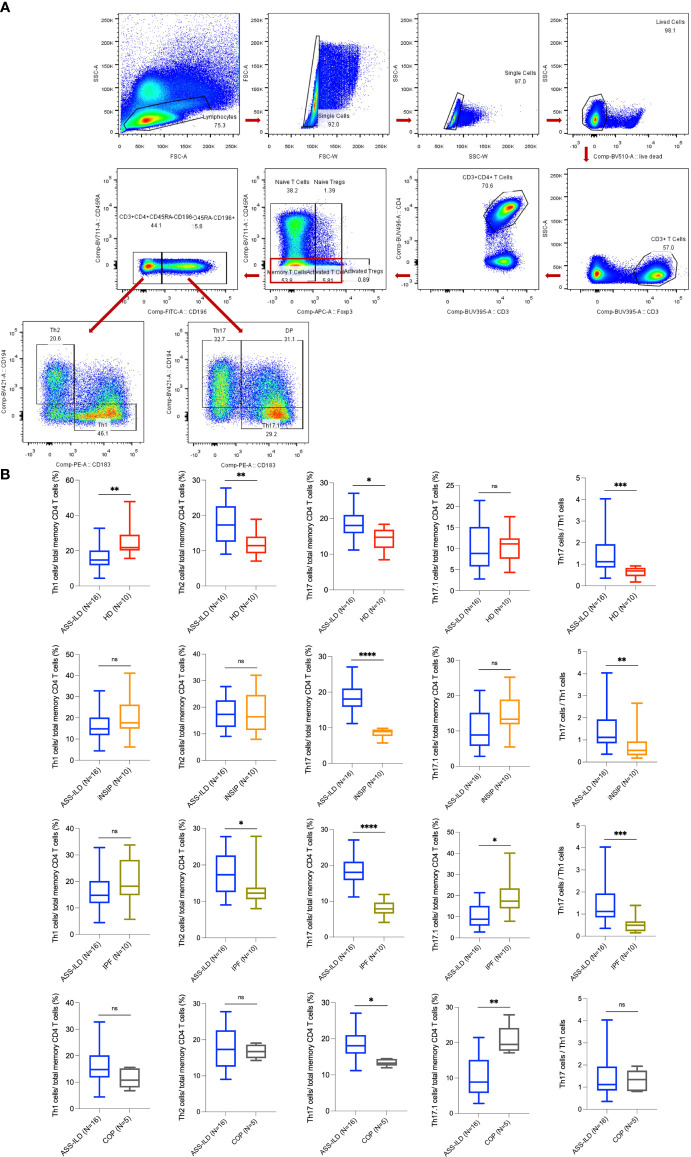
Flow cytometry experiments. **(A)** Representative flow cytometry analyses of PBMCs from one patient with ASS-ILD. **(B)** Percentages of Th1, Th2, Th17, and Th17.1 cells in total memory CD4 T cells among PBMCs of 16 patients with ASS-ILD, 10 patients with iNSIP, 10 patients with IPF, 5 patients with COP, and 10 HDs, respectively. And the ratio of Th17 cells to Th1 cells. The results were calculated by the Mann-Whitney test, respectively. The results were considered significant when a two-sided p-value was <0.05. PBMC, peripheral blood mononuclear cell; ASS-ILD, anti-synthetase syndrome-associated interstitial lung disease; iNSIP, idiopathic non-specific interstitial pneumonia; IPF, idiopathic pulmonary fibrosis; COP, cryptogenic organizing pneumonia; HD, healthy donor. *p < 0.05; **p < 0.01; ***p < 0.001; ****p < 0.0001; ns, not statistically significant.

To gain insights into the differential transcriptomic changes in T cells between patients with ASS-ILD and HDs, we compared their expression profiles of T cells. We observed that DEGs upregulated in patients with ASS-ILD were involved in processes including responses to type I and II interferons, cellular response to interleukin-1, cellular response to tumor necrosis factor, and NF-kappaB signaling using GO analysis (**Figure 6A**). We also did Kyoto Encyclopedia of Genes and Genomes (KEGG) pathway analysis. Of note, Th1, Th2, and Th17 cell differentiation signaling pathways were enriched in T cells, indicating the involvement of Th1, Th2, and Th17 cells in the pathogenesis of ASS-ILD (**Figure 6B**). Enriched genes associated with Th1, Th2, and Th17 cell differentiation signaling pathways included HLA-DRB5, HLA-DQA1, IL2RG, HLA-DPA1, HLA-DPB1, HLA-DRB1, and HLA-DRA. The expressions of genes associated with the differentiation of CD4 T cells were mapped (**Figures 6C, D** **)**. To validate these findings, we performed flow cytometry experiments to compare the differences in Th1 proportions, Th2 proportions, Th17 cell proportions, Th17.1 proportions, and ratios of Th17 cells to Th1 cells between patients with ASS-ILD and patients with idiopathic interstitial pneumonia (IIP, including iNSIP, COP, and IPF), and HDs, respectively. The clinical manifestations, laboratory features, chest HRCT patterns, and PFT parameters of recruited patients and HDs were detailed in **Supplementary Tables 3** and **4**. The results indicated that the proportion of Th1 cells among total memory CD4 T cells was lower in patients with ASS-ILD than that in HDs; the proportion of Th2 cells among total memory CD4 T cells was higher in patients with ASS-ILD than that in patients with IPF, and HDs, respectively; the proportion of Th17 cells among total memory CD4 T cells was higher in patients with ASS-ILD than that in HDs, patients with iNSIP, patients with COP, and patients with IPF, respectively; the proportions of Th17.1 cells among total memory CD4 T cells were similar in patients with ASS-ILD and HDs; the ratio of Th17 cells to Th1 cells was higher in patients with ASS-ILD than that in patients with iNSIP, patients with IPF, and HDs, respectively (**Figures 7A, B** **)**.

### Features of B Cell Subsets in Patients With ASS-ILD

**Figure 8:**
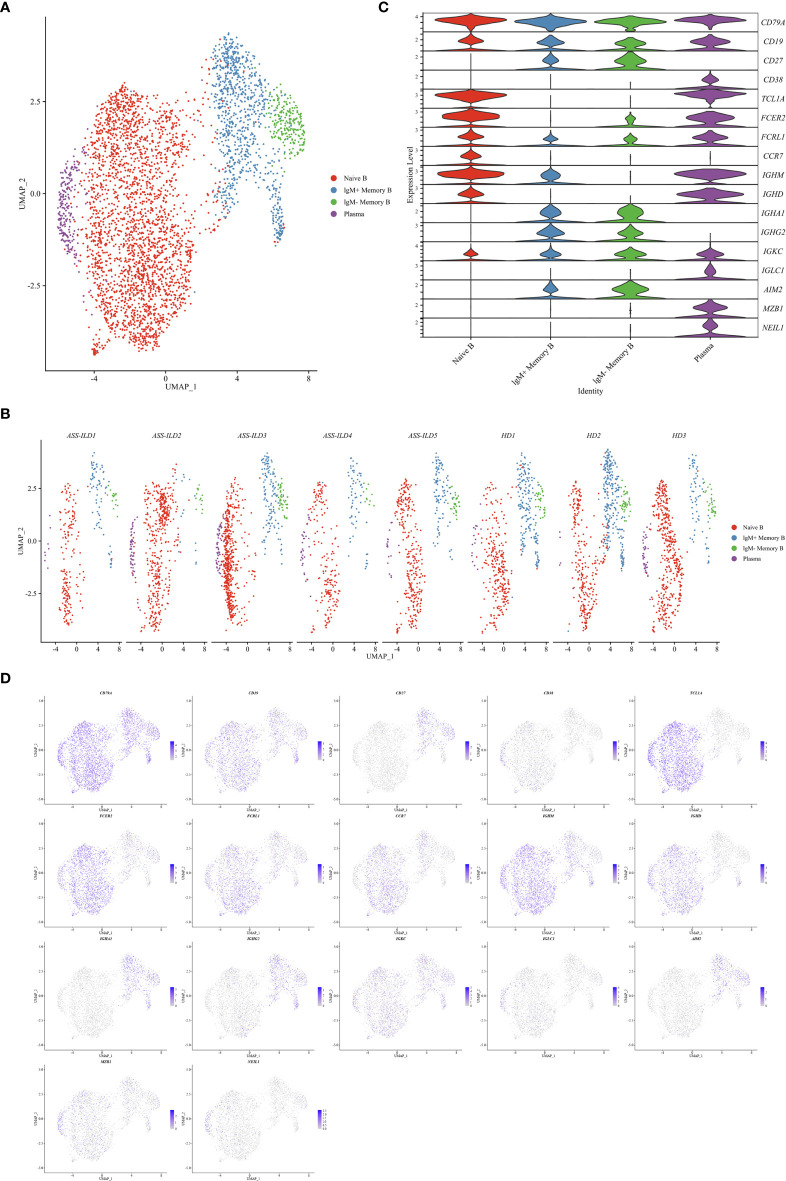
Immunological features of B cell subsets. **(A)** UMAP visualization of 4709 B cells. **(B)** Annotating condition of each patient with ASS-ILD and HD. **(C)** Violin plot showing the expression distribution of selected marker genes in the 4 B cell subsets. The rows represent selected marker genes and the columns represent clusters with the same color as in **(A)**. **(D)** Expression of canonical marker genes for 4 B cell subsets as represented in the UMAP plot. Data are colored according to expression levels and the legend is labeled in log scale. UMAP, uniform manifold approximation and projection; ASS-ILD, anti-synthetase syndrome-associated interstitial lung disease; HD, healthy donor.

To characterize changes of different B subsets, we sub-clustered B cells into 8 subsets and those with similar B cell markers were categorized together (**Supplementary Figures 6A–H**). Then we identified 4 subsets of B cells based on the expression of canonical B cell markers (**Figures 8A, B** **)**. We identified naïve B lymphocytes (CD79A+CD19+IGHD+), IgM+ memory B lymphocytes (CD79A+CD19+CD27+IGHM+), IgM- memory B lymphocytes (CD79A+CD19+CD27+IGHM-), and plasma (CD38+MZB1+) (**Figures 8C, D** **)**. No significant differences in cell proportions were seen in B cells (**Supplementary Figures 7A, B**).

**Figure 9:**
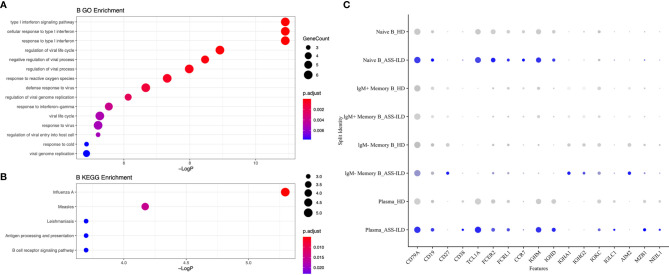
Enrichment analyses for B cells. **(A)** GO enrichment analysis of upregulated genes for B cells. **(B)** KEGG pathway enrichment analysis of upregulated genes for B cells. **(C)** Dot plot depicting average expression and percentage of expressed cells of selected marker genes in each labeled B cell subset. GO, Geno Ontology; KEGG, Kyoto Encyclopedia of Genes and Genomes.

To investigate the differential transcriptomic changes in B cells between patients with ASS-ILD and HDs, we then compared their expression profiles of B cells. We observed that DEGs upregulated in patients with ASS-ILD were involved in processes including responses to type I and II interferons, regulation of viral life cycle, and response to reactive oxygen species (**Figure 9A**). We also performed KEGG pathway analysis, Influenza A, Measles, antigen processing and presentation were enriched in B cells (**Figure 9B**). A dot plot showed the average expression and percentage of expressed cells of selected marker genes in each labeled B cell subset (**Figure 9C**).

## Discussion

Currently, the management of patients with ASS-ILD relies on glucocorticoids combined with immunosuppressant or biological agents (10, 11). However, the pathogenesis of ASS remains to be clearly elucidated and specific target therapies are still lacking. Several studies have shown the features of innate and adaptive immune responses, which have helped us understand the pathogenic immune responses of ASS-ILD (13, 20, 21). It is evident that both the innate and adaptive immune responses contribute to the breakdown of immune tolerance to self-antigen in autoimmune diseases, and adaptive immune cells including T and B cells are primary contributors. In this study, we aimed to profile the immunological landscape in PBMCs from patients with ASS-ILD at single-cell resolution and investigate the roles of different CD4 T cells in this condition.

First, patients with ASS-ILD showed upregulated interferon responses among NK cells, monocytes, T cells, and B cells in PBMCs, which is consistent with previous studies (19, 21). In autoimmune diseases, the presence of persistent production of both type I and type II interferons has been well established (22). The most well-defined type I interferons are interferon *α* and interferon *β*, and type II interferon only includes interferon *γ* (23). Interferon α, β, and γ induce the expression of transcription factors, especially canonical interferon-stimulating genes (ISGs), by activating different JAK-STAT pathways (23). Targeting therapies against JAK-STAT pathways have been developed and some of them were used in clinics as an alternative to biological therapies. Tofacitinib is an oral small molecule targeting all JAKs but preferentially inhibits JAK1 and JAK3, and it is the first approved JAK inhibitor for rheumatic arthritis (24). Two prospective clinical trials reported that tofacitinib is effective in treating amyopathic dermatomyositis-associated ILD (25, 26). The efficacy and safety of tofacitinib in patient with ASS-ILD have only been reported in a case report (27). There are more small-molecule compounds targeting JAKs in development and different JAK inhibitors show different specificities (28, 29). Tofacitinib and other JAK inhibitors may be of benefit in treating ASS-ILD, but further investigations are still warranted to evaluate their efficacy and safety profiles.

Second, the proportion of naïve CD8 T cells in patients with ASS-ILD measured by scRNA-seq seemed lower than that in HDs, while the proportion of CD8 TEM cells seemed higher, but no significant differences were detected. We further compared the ratio of CD8 TEM to CD8 Naïve, patients with ASS-ILD showed a significantly higher proportion than that in HDs. CD8 TEM could be categorized into 3 subsets according to specific transcriptional features in this study: CD8 TEM GZMK, CD8 TEM GNLY, CD8 TEM TRAV 27. GZMK, GNLY, TRAV 27 was specifically up-regulated in these three subsets, respectively. GZMK and GNLY are different lytic granules (30), TRAV 27 gene encodes V region of the variable domain of T cell receptor *α* chain to participate in antigen recognition (31). We only found significant differences in the ratio CD8 TEM GNLY to CD8 Naïve between patients with ASS-ILD and HDs. Current researches have reported the correlations between GNLY or CD8 T cells and several autoimmune diseases, such as differential expressions of GNLY in rheumatic arthritis and healthy state, an association of GNLY expressions in CD8 cells and steroid resistance in polymyositis, an association of disease flares and proportions of activated CD8 T cells in systemic lupus erythematosus (32–34). Our study showed that CD8 T cells in the blood of patients with ASS-ILD were skewed towards an activated phenotype characterized by high expression of GNLY. Further investigations into whether CD8 T cells infiltrate and mediate damages in target organs might help to better illustrate the role of CD8 T cells in ASS-ILD. And the role of GNLY and other cytolytic granules in CD8 T cells is worth exploring to disclose various functions of CD8 T cells.

Third, Th1, Th2, and Th17 cell differentiation signaling pathways were enriched in T cells in this study. Using scRNA-seq here was unable to directly compare the differences in the compositions of Th1, Th2, and Th17 cells between patients with ASS-ILD and HDs. We performed validation experiments using flow cytometry, which was consistent with our scRNA-seq results. In consideration for overlapping chest radiological manifestations between ASS-ILD and IIP (especially NSIP, COP, and IPF), we enrolled patients with IIP as disease controls in validation experiments. The proportions of Th2 cells and Th17 cells were higher in patients with ASS-ILD, and the proportion of Th1 cells was lower. Although hallmark pathogenic conditions associated with Th2 cells are allergy and asthma (35), it is worth mentioning that Th2 dominant autoimmune diseases are present, like multiple sclerosis (36). Besides a shift to a predominant Th2 response has been reported to lead to chronic inflammation and pulmonary fibrosis in sarcoidosis and hypersensitivity pneumonitis, as well as airway fibrosis induced by asthma (37–39). It is worth thinking whether the dynamic changes of Th2 cells are related with the fibrotic evolution of ILD in ASS. Establishing the risk factors for fibrotic ASS-ILD could provide us with the opportunity to early identify and intervene the progression of pulmonary fibrosis. Th17 cells have been demonstrated to play important roles in several autoimmune diseases and chronic respiratory diseases, such as rheumatic arthritis, psoriasis, multiple sclerosis, asthma, and sarcoidosis (18, 40, 41).Th17 cells are characterized by the hallmark cytokine of IL-17A, and have a critically important role in the induction of multiple autoimmune disorders (40). Th17 cells display remarkable plasticity, which is also closely associated with their pathogenicity (42, 43). Here we used chemokine receptors to define different subsets of CD4 T cells and found the increased numbers of Th17 cells in this study. It remains uncertain how Th17 cells with a high grade of plasticity function in this condition. It is not only important to evaluate Th17 cell frequency in this condition, but also to explore the proinflammatory capacity of Th17 cells. Thus how Th17 cells function in this condition is worthy of exploration.

Fourth, GO analysis for NK cells showed the enrichment of antigen processing and presentation of peptide antigen *via* MHC II, which needs to be interpreted carefully. NK cells are not professional antigen-presenting cells (APCs), and they are important members of the innate immune system to directly eliminate virus-infected cells and tumor cells through their natural cytotoxic capacity without prior priming (44). Genes enriched for antigen processing and presentation mainly included human leukocyte antigen (HLA) -DPB1, HLA-DPA1, HLA-DQA1, HLA-DRB1, and HLA-DRB5, all these enriched genes are classical HLA II molecules. HLA-DR-expressing NK cells have been reported to be detected in the blood and tissues in healthy individuals, and their functions are associated with enhanced production of proinflammatory cytokines, degranulation, and proliferation upon stimuli (44). *In vitro*, HLA-DR-expressing NK cells pre-activated with cytokines have been reported to induce nonspecific activation and partial differentiation of CD4 T cells in previous publications (45, 46). However, how NK cells process and present antigen is unclear and *in vivo* validation experiments are still lacking. The ability of antigen processing and presentation remains to be determined for NK cells. Type I interferons are well-known powerful regulators of NK cell activation (47), and HLA-DR expressing NK cells are reported to display profound ability to induce secretion of interferon *γ* (48). Functional divergence of NK cells from patients with ASS-ILD and HDs exists based on our analysis. We speculate that DEGs associated with classical HLA II molecules could be the effect of interferons in this condition. Nevertheless, more analyses are needed to verify the enriched functions profiled by scRNA-seq analyses.

There are several limitations in our study. First, the sample size is comparatively small. Second, we don’t have tissue samples, limiting our interpretation of cellular interchange between target organs and peripheral compartments. Third, longitudinal follow-up studies are needed to further elucidate the pathogenic roles of upregulated interferon responses, altered CD8 T cell homeostasis, and involvement of differentiation signaling pathways of CD4 T cells in the pathogenesis of ASS-ILD. Fourth, scRNA-seq of PBMCs is a powerful tool for visualization of peripheral cellular landscape, but it remains to be interpreted carefully owing to technical limitations and heterogeneity of algorithms for data analyses in different researches. Nevertheless, our research reveals peripheral immune responses in patients with ASS-ILD, and may provide foundations for further exploration into the pathogenesis of this condition.

In conclusion, our study reveals that patients with ASS-ILD are characterized by upregulated interferon responses, altered CD8 T cell homeostasis, and involvement of differentiation pathways for different subsets of CD4+ T cells. And the proportions of Th17 cells and Th2 cells are increased in patients with ASS-ILD than those in HDs based on the expression patterns of chemokine receptors through flow cytometry, while the proportion of Th1 cells is decreased. Further studies with longitudinal samples from more patients and investigations into cellular interchange between target organs and peripheral immune cells are still warranted to advance our understanding of the pathogenesis, which may pave the way for novel therapeutic interventions against ASS-ILD.

## Data Availability Statement

The datasets presented in this study can be found in online repositories. The names of the repository/repositories and accession number(s) can be found below: NCBI [accession: GSE190510].

## Ethics Statement

The studies involving human participants were reviewed and approved by the ethics committee of the China-Japan Friendship Hospital, China (2019-123-K85). The patients/participants provided their written informed consent to participate in this study.

## Author Contributions

CZ, HD, and CW contributed equally to this artricle and are joint corresponding authors. LilZ, ZC, SW, HD, and CW designed the study. LilZ was responsible for the data collection. ZC was responsible for the data analysis, with support from CZ and LF. LilZ and ZC interpreted the data and prepared the first draft. LilZ, ZC, SW, CZ, LF, YR, BX, JG, HD, CW revised the draft. SX, LZ, and LM were involved in identification of patients with ASS-ILD or IIP. LilZ, ZC, HD, and CW had full access to all of the data in the study and took responsibility for the integrity of the data and the accuracy of the data analyses. The corresponding authors attest that all listed authors meet authorship criteria and that no others meeting the criteria have been omitted. All authors contributed to the article and approved the submitted version.

## Funding

This study was financially supported by Chinese Academy of Medical Sciences Innovation Fund for Medical Sciences (2018-I2M-1-001), the National Key Technologies R&D Programme Precision Medicine Research (2016YFC0901101) and the National Natural Science Foundation of China (81870056). 

## Conflict of Interest

LF is employed by DataCanvas Technology Co., Ltd.

The remaining authors declare that the research was conducted in the absence of any commercial or financial relationships that could be construed as a potential conflict of interest.

## Publisher’s Note

All claims expressed in this article are solely those of the authors and do not necessarily represent those of their affiliated organizations, or those of the publisher, the editors and the reviewers. Any product that may be evaluated in this article, or claim that may be made by its manufacturer, is not guaranteed or endorsed by the publisher.
