# Supplementary material for: Single-Cell Transcriptomics Reveals Peripheral Immune Responses in Anti-Synthetase Syndrome-Associated Interstitial Lung Disease
Source: Front Immunol. 2022 Feb 17;13:804034. doi: 10.3389/fimmu.2022.804034 (PMC8891123; doi:10.3389/fimmu.2022.804034)
Supplement: Supplementary file 1 [file DataSheet_1.pdf]

Supplementary Material

Supplementary Table 1 Clinical manifestations and laboratory features of patients with ASS-ILD and HDs in the single-cell RNA sequencing cohort.

Parameters	ASS-ILD (n=5)	HD (n=3)
Age, years	56±9	52±4
Female, n	5	3
Duration of symptoms before diagnosis, months	5±2	-
Clinical characteristics, n		-
Myositis	1	
ILD	5	
Mechanic's hands	5	
Fever	0	
Arthritis	0	
Raynaud's phenomenon	0	
Gottron's sign	0	
ASA subtype, n		-
EJ	3	
PL-7	2	
White blood cells, × 10^9/L	5.6 (4.5-6.9)	NA
Neutrophils, × 10^9/L	3.4 (2.2-4.6)	NA

Lymphocytes, × 10^9/L	1.6 (1.3-2.0)	NA
Alanine aminotransferase, IU/L	19 (16-22)	NA
Aspartate aminotransferase, IU/L	19(17-25)	NA
Lactate dehydrogenase, IU/L	163 (155-254)	NA
Creatine kinase, IU/L	43 (34-146)	NA
Creatinine, umol/L	57 (46-68)	NA
Erythrocyte sedimentation rate, mm/h	12 (6-16)	NA
Serum ferritin, ng/mL	77 (19-306)	NA
Positive anti-nulcear antibody (titer ≥1:80), n	5	NA
KL-6, U/ml	1009 (650-3150)	NA
Positive Ro-52, n	3	NA
PaO2/FiO2 ratio, mmHg	419 (395-426)	NA

ASS-ILD, anti-synthetase syndrome-associated interstitial lung disease; HD, healthy donor; ASA, anti-tRNA synthetase antibody; NA, not available.

Supplementary Table 2 Chest HRCT patterns and PFT parameters of patients with ASS-ILD in the single-cell RNA sequencing cohort.

Parameters	ASS-ILD
Chest HRCT pattern, n	

NSIP+OP	4
NSIP	1
FVC %pred	72 (70-91)
FEV1 % pred	70 (67-89)
FEV1/FVC, %	80 (79-85)
TLC % pred	68 (65-84)
DLCO % pred	65 (55-79)

HRCT, high-resolution computed tomography; PFT, pulmonary function test, ASS-ILD, anti-synthetase syndrome-associated interstitial lung disease; NSIP, non-specific interstitial pneumonia; OP, organizing pneumonia; FVC, forced vital capacity; FEV1, forced expiratory volume in one second; TLC, total lung capacity; DLCO, diffusing capacity of carbon monoxide.

Supplementary Table 3 Clinical manifestations and laboratory features of patients with ASS-ILD, patients with IIP, and HDs in the flow cytometry cohort.

Parameters	ASS-ILD (n=16)	iNSIP (n=10)	COP (n=5)	IPF (n=10)	HD (n=10)	p value
Age, years	57±12	59±6	55±13	58±6	50±7	0.068
Female, n	13*	4	2	2	9	0.002
Duration of symptoms before diagnosis, months	4±3	-	-	-	-	-
Clinical characteristics, n					-	-
Myositis	3	0	0	0		
ILD	16	10	5	10		

Mechanic's hands	12	0	0	0		
Fever	1	0	0	0		
Arthritis	3	0	0	0		
Raynaud's phenomenon	0	0	0	0		
Gottron's sign	2	0	0	0		
ASA subtype, n		-	-	-	-	-
EJ	5					
PL-7	7					
Jo-1	3					
PL-12	1					
White blood cells, × 10^9/L	6.1 (5.4-7.3)	6.6 (5.6-7.5)	5.8 (4.7-10.2)	7.1 (5.7-8.5)	NA	-
Neutrophils, × 10^9/L	3.7 (2.7-4.7)	3.5 (3.0-4.0)	3.7 (2.7-7.1)	4.3 (2.9-5.5)	NA	-
Lymphocytes, × 10^9/L	1.8 (1.3-2.0)	2.3 (1.9-2.8)	1.4 (1.3-2.4)	2.2 (1.5-2.6)	NA	-
Alanine aminotransferase, IU/L	20 (15-30)	20 (16-24)	25 (21-53)	22 (16-34)	NA	-
Aspartate aminotransferase, IU/L	21 (18-24)	19 (17-22)	19 (18-30)	18 (16-28)	NA	-
Lactate dehydrogenase, IU/L	211 (167-256)	191 (180-236)	185 (160-206)	216 (177-252)	NA	-

Creatine kinase, IU/L	64 (41-128)	68 (63-98)	67 (34-102)	57 (41-93)	NA	-
Creatinine, umol/L	56 (49-68)	60 (56-67)	62 (47-81)	71 (65-79)	NA	-
Erythrocyte sedimentation rate, mm/h	10 (5-19)	11 (3-21)	10 (6-11)	16 (5-25)	NA	-
Serum ferritin, ng/mL	141 (28-249)	NA	NA	NA	NA	-
CD4:CD8	1.49(0.81-2.45)	1.5(0.70-1.93)	1.51(1.38-1.69)	1.04(0.75-1.48)	NA	-
Positive anti-nuclear antibody (titer ≥1:80, n	7	3	0	3	NA	-
KL-6, U/ml	1230 (697-2142)	990 (401-3467)	708 (252-1163)	863 (521-2013)	NA	-
Positive Ro-52, n	7	0	1	0	NA	-
PaO2/FiO2 ratio, mmHg	421 (381-432)	419 (388-433)	367 (345-429)	395 (348-441)	NA	-

ASS-ILD, anti-synthetase syndrome-associated interstitial lung disease; IIP, idiopathic interstitial pneumonia; HD, healthy donor; iNSIP, idiopathic non-specific interstitial pneumonia; COP, cryptogenic organizing pneumonia; IPF, idiopathic pulmonary fibrosis; ASA, anti-tRNA synthetase antibody; *, no significant difference between ASS-ILD and HDs; NA, not available.

Supplementary Table 4 Chest HRCT patterns and PFT parameters of patients with ASS-ILD and IIP in the flow cytometry cohort.

Parameters	ASS-ILD	iNSIP	COP	IPF

	(n=16)	(n=10)	(n=5)	(n=10)
Chest HRCT pattern, n	NSIP: 5<br>NSIP+OP: 9<br>OP: 2	NSIP: 10	OP: 5	Probable UIP: 3<br>UIP: 7
FVC %pred	69 (64-94)	83 (72-92)	81 (55-108)	78 (65-89)
FEV1 % pred	72 (63-88)	85 (77-96)	77 (55-102)	82 (69-100)
FEV1/FVC, %	84 (78-85)	83 (77-90)	79 (75-83)	84 (83-87)
TLC % pred	70 (57-75)	71 (59-97)	76 (51-101)	68 (54-75)
DLCO % pred	62 (50-68)	56 (42-69)	65 (51-73)	54 (31-69)

HRCT, high-resolution computed tomography; PFT, pulmonary function test; ASS-ILD, anti-synthetase syndrome-associated interstitial lung disease; IIP, idiopathic interstitial pneumonia; iNSIP, idiopathic non-specific interstitial pneumonia; COP, cryptogenic organizing pneumonia; IPF, idiopathic pulmonary fibrosis; UIP, usual interstitial pneumonia; FVC, forced vital capacity; FEV1, forced expiratory volume in one second; TLC, total lung capacity; DLCO, diffusing capacity of carbon monoxide.

A

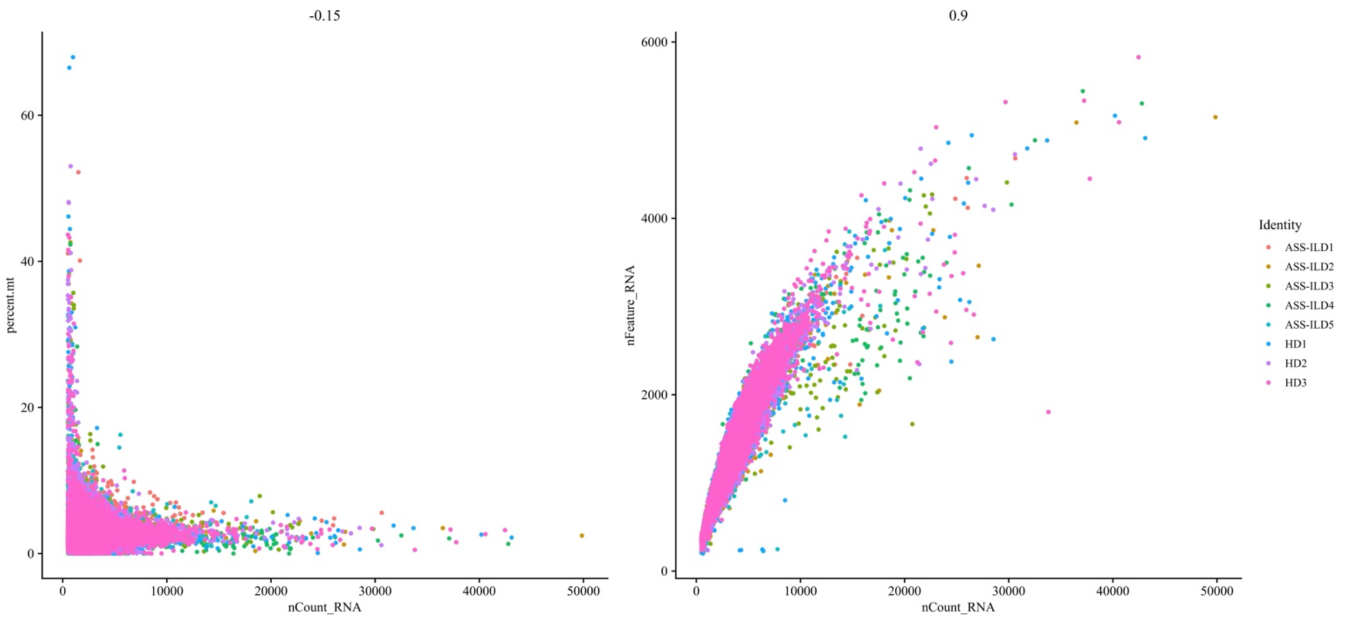

B

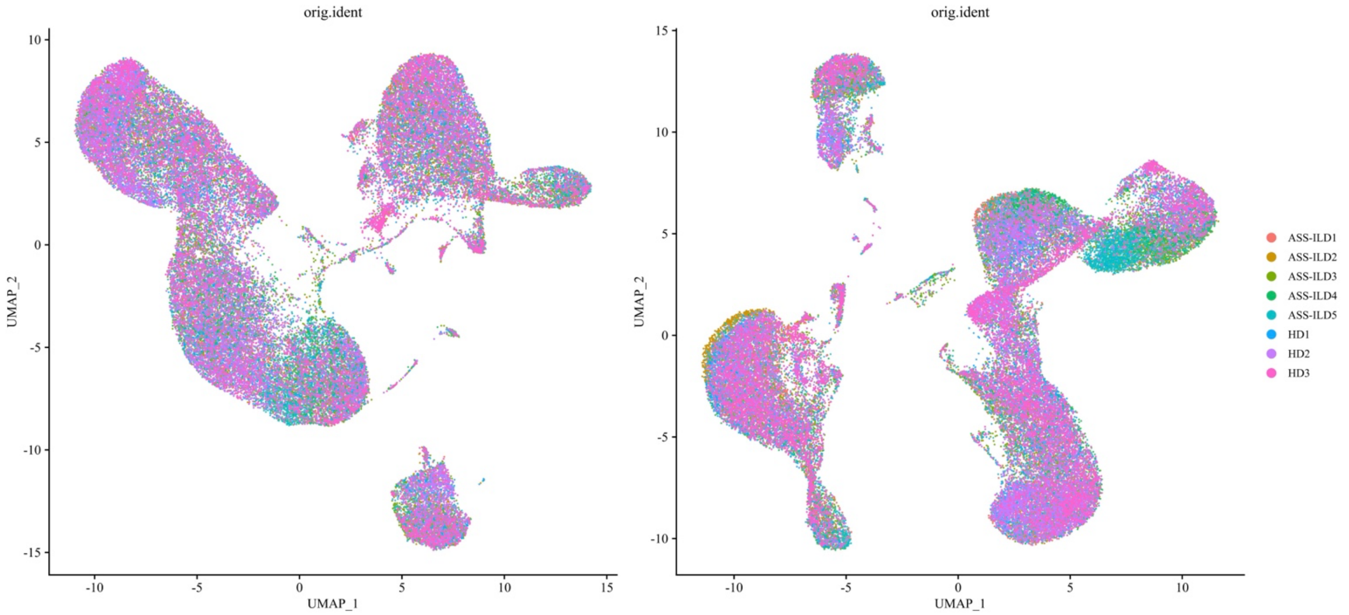

Supplementary Figure 1 Quality control and batch effect correction. (A) Plots of quality control. **(B)** UMAP visualization before (right) and after (left) batch effect correction. ASS-ILD, anti-synthetase syndrome-associated interstitial lung disease; HD, healthy donor; UMAP, uniform manifold approximation and projection.

A

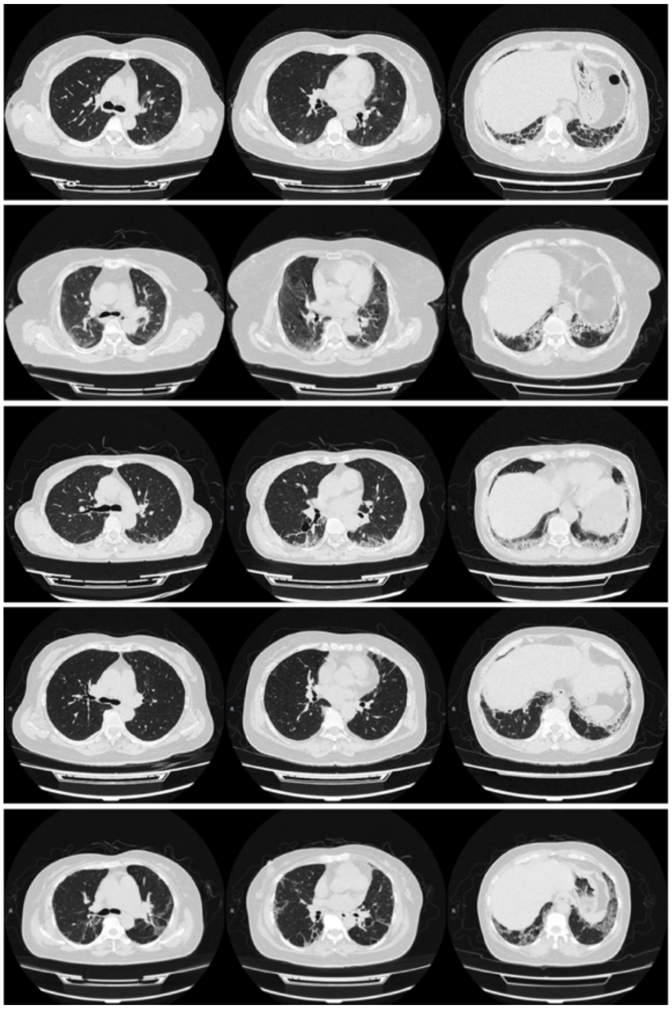

B

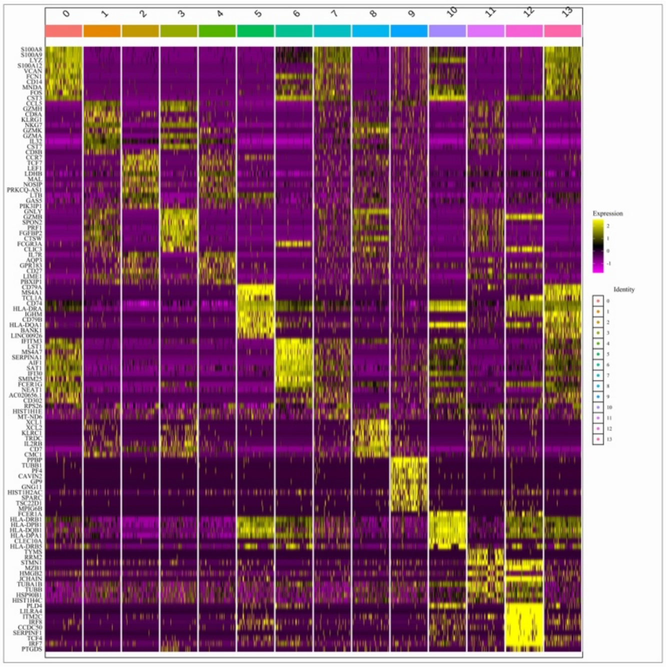

C

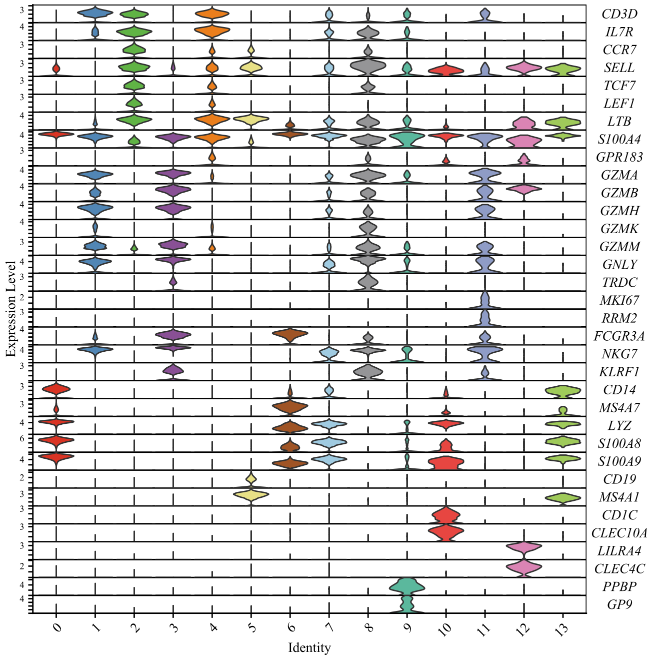

Supplementary Figure 2 Chest HRCT images of 5 patients, heatmap and violin plot of all clusters. (A) Chest HRCT images of 5 enrolled patients with ASS-ILD showing bilateral ground-glass opacities, reticulation, and focal consolidation. **(B)** Heatmap showing the up- and down-regulated genes in all clusters. **(C)** Violin plot depicting the expression distribution of selected marker genes in

all clusters. The rows represent selected marker genes and the columns represent clusters. Cluster 7 highly expressed CD3D (T cell marker) and CD14 (Monocyte marker), and cluster 13 highly expressed CD14 (Monocyte marker) and MS4A1 (B cell marker). Cluster 7 and cluster 13 were regarded as doublets. HRCT, high-resolution computed tomography; ASS-ILD, anti-synthetase syndrome-associated interstitial lung disease.

A

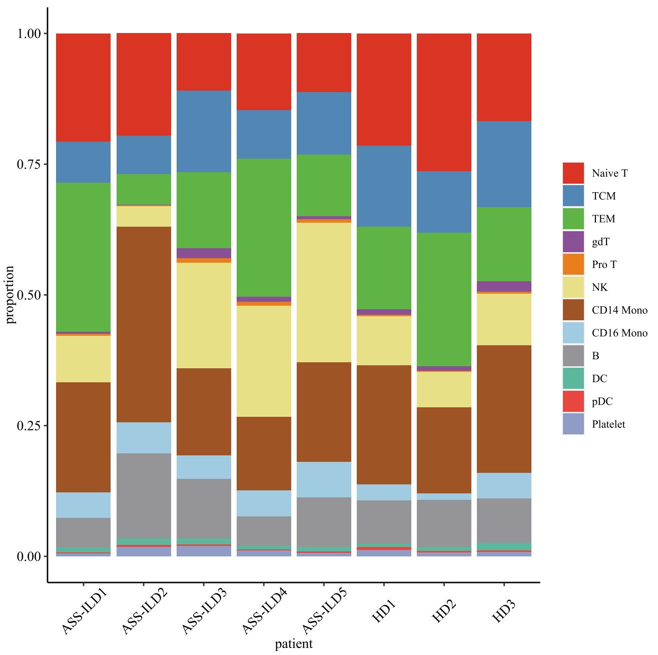

B

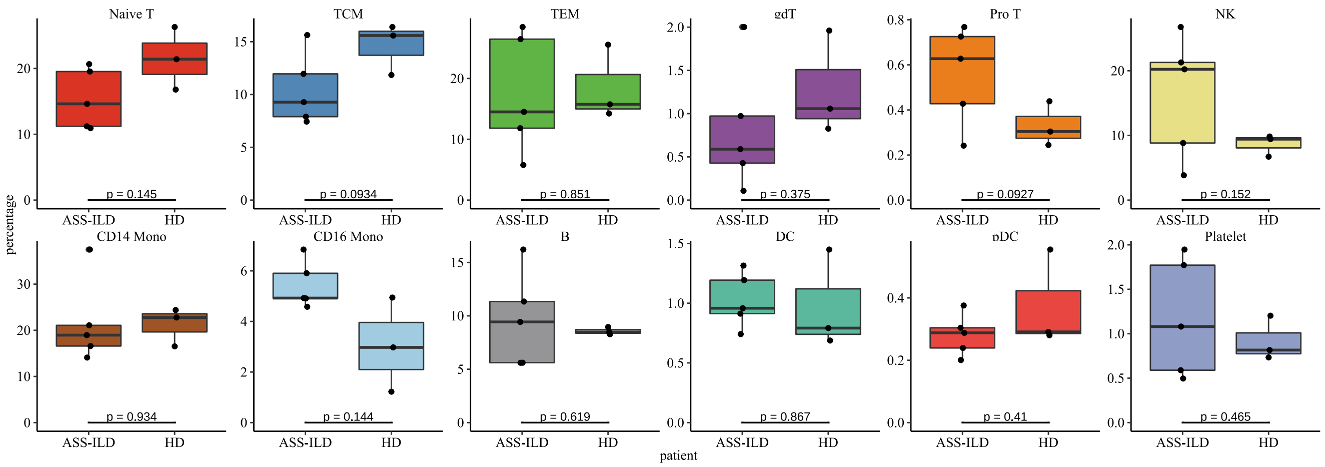

Supplementary Figure 3 Bar plots showing cell proportions. (A) Bar plot depicting cell compositions of each sample. (**B**) Bar plot depicting average cell percentage of each cluster between patients with ASS-ILD and HDs. Error bars represent mean ± s.e.m. for 5 patients and 3 HDs. TCM,

central memory T lymphocyte; TEM, effector memory T lymphocyte; pro T, proliferative T cell; NK, natural killer cell; mono, monocyte; DC, dendritic cell; pDC, plasmacytoid DC; ASS-ILD, anti-synthetase syndrome-associated interstitial lung disease; HD, healthy donors; s.e.m., standard error of mean.

A

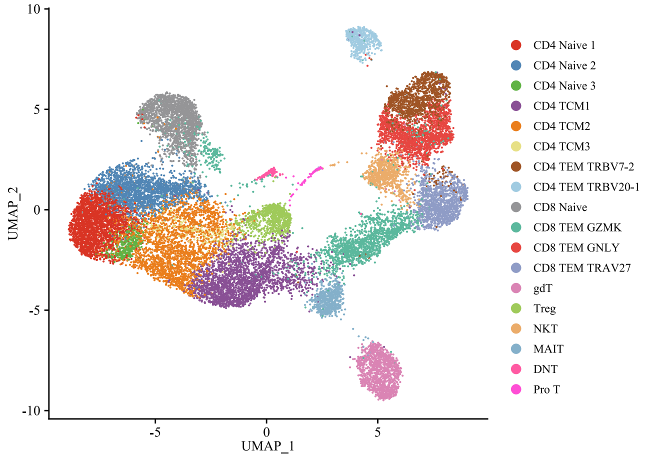

B

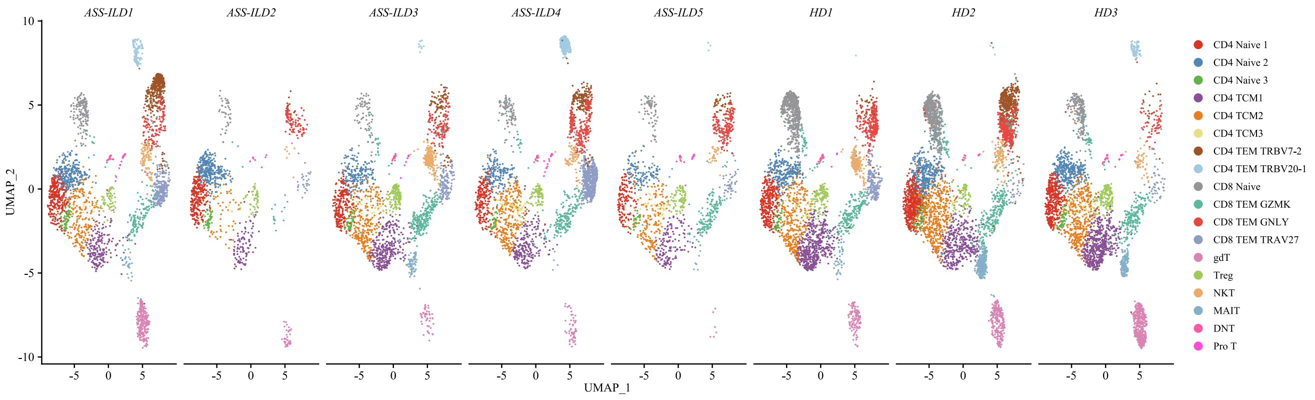

C

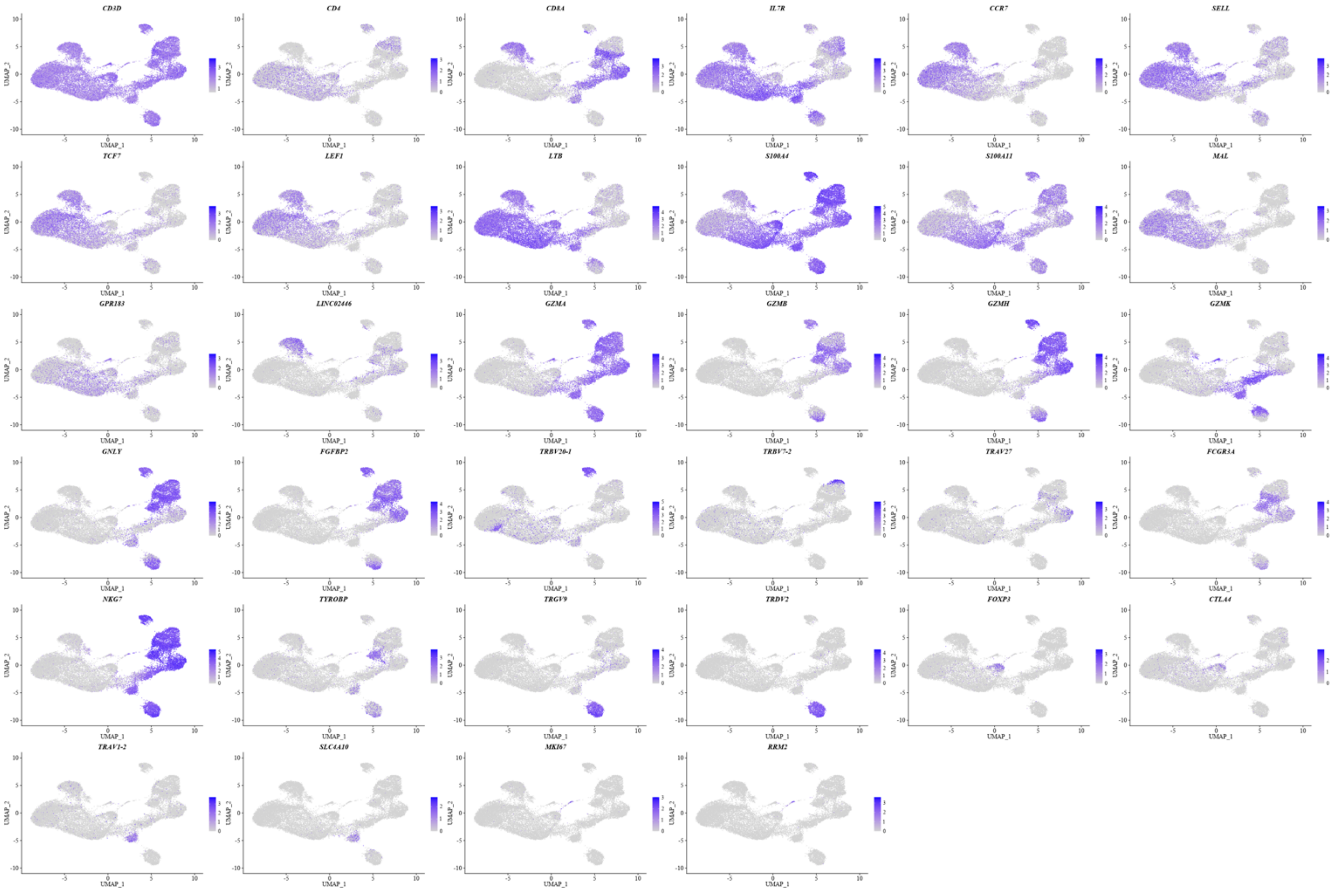
CD3D
CD4
CD8A
IL7R
CCR7
SELL
TCF7
LEF1
LTB
S100A4
S100A11
MAL
GPR183
LINC02446
GZMA
GZMB
GZMH
GZMK
GNLY
FGFBP2
TRBV20-1
TRBV7-2
TRAV27
FCGR3A
NKG7
TYROBP
TRGV9
TRDV2
FOXP3
CTLA4
TRAV1-2
SLC4A10
MKI67
RRM2
UMAP_1
UMAP_2

D

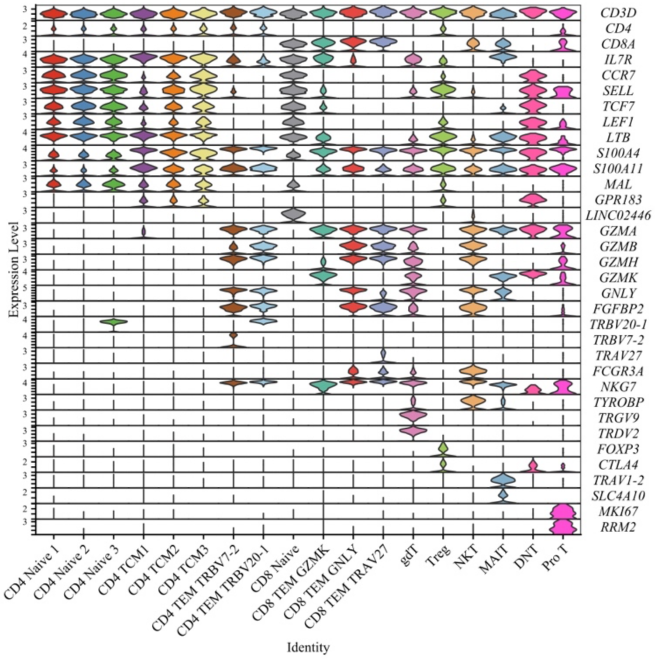
Expression Level
CD3D
CD4
CD8A
IL7R
CCR7
SELL
TCF7
LEF1
LTB
S100A4
S100A11
MAL
GPR183
LINC02446
GZMA
GZMB
GZMH
GZMK
GNLY
FGFBP2
TRBV20-1
TRBV7-2
TRAV27
FCGR3A
NKG7
TYROBP
TRGV9
TRDV2
FOXP3
CTLA4
TRAV1-2
SLC4A10
MKI67
RRM2
CD4 Naive 1
CD4 Naive 2
CD4 Naive 3
CD4 TCM1
CD4 TCM2
CD4 TCM3
CD4 TEM TRBV7-2
CD4 TEM TRBV20-1
CD8 Naive
CD8 TEM GZMK
CD8 TEM GNLY
CD8 TEM TRAV27
gdT
Treg
NKT
MAIT
DNT
Pro T
Identity

E

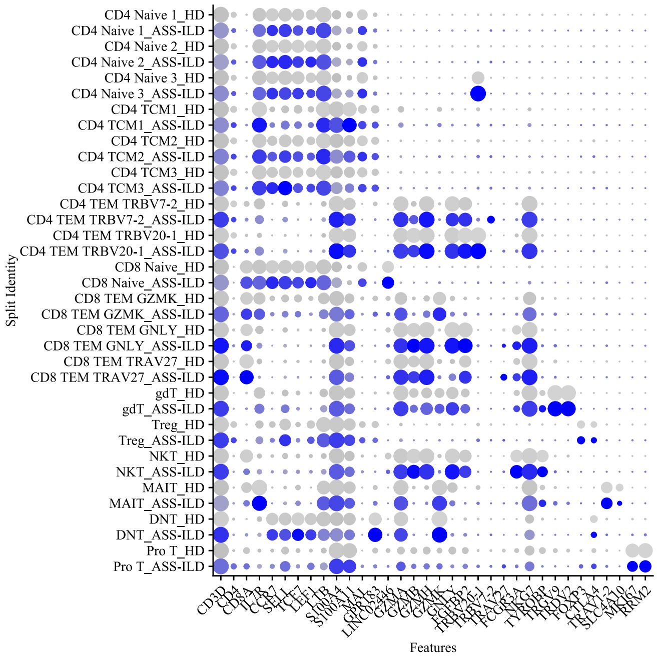
CD4 Naive 1_HD
CD4 Naive 1_ASS-ILD
CD4 Naive 2_HD
CD4 Naive 2_ASS-ILD
CD4 Naive 3_HD
CD4 Naive 3_ASS-ILD
CD4 TCM1_HD
CD4 TCM1_ASS-ILD
CD4 TCM2_HD
CD4 TCM2_ASS-ILD
CD4 TCM3_HD
CD4 TCM3_ASS-ILD
CD4 TEM TRBV7-2_HD
CD4 TEM TRBV7-2_ASS-ILD
CD4 TEM TRBV20-1_HD
CD4 TEM TRBV20-1_ASS-ILD
CD8 Naive_HD
CD8 Naive_ASS-ILD
CD8 TEM GZMK_HD
CD8 TEM GZMK_ASS-ILD
CD8 TEM GNLY_HD
CD8 TEM GNLY_ASS-ILD
CD8 TEM TRAV27_HD
CD8 TEM TRAV27_ASS-ILD
gdT_HD
gdT_ASS-ILD
Treg_HD
Treg_ASS-ILD
NKT_HD
NKT_ASS-ILD
MAIT_HD
MAIT_ASS-ILD
DNT_HD
DNT_ASS-ILD
Pro T_HD
Pro T_ASS-ILD
Split Identity
Features

F

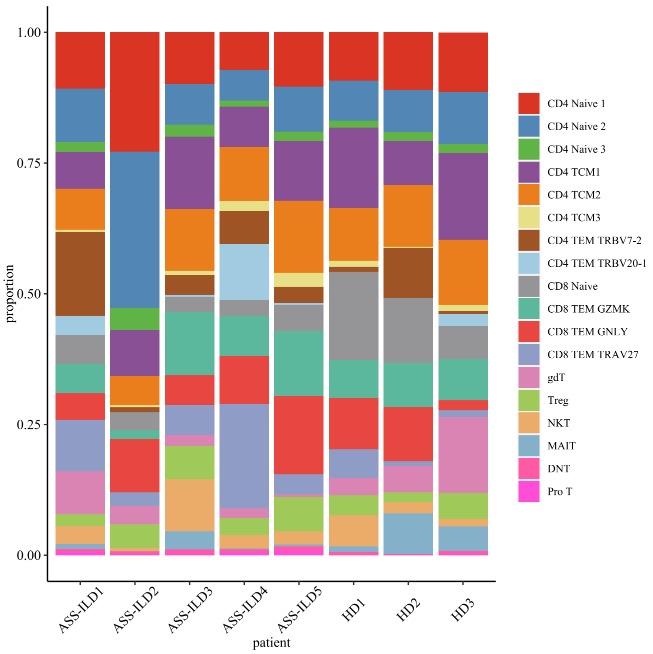
1.00
0.75
0.50
0.25
0.00
proportion
ASS-ILD1
ASS-ILD2
ASS-ILD3
ASS-ILD4
ASS-ILD5
HD1
HD2
HD3
patient
CD4 Naive 1
CD4 Naive 2
CD4 Naive 3
CD4 TCM1
CD4 TCM2
CD4 TCM3
CD4 TEM TRBV7-2
CD4 TEM TRBV20-1
CD8 Naive
CD8 TEM GZMK
CD8 TEM GNLY
CD8 TEM TRAV27
gdT
Treg
NKT
MAIT
DNT
Pro T

G

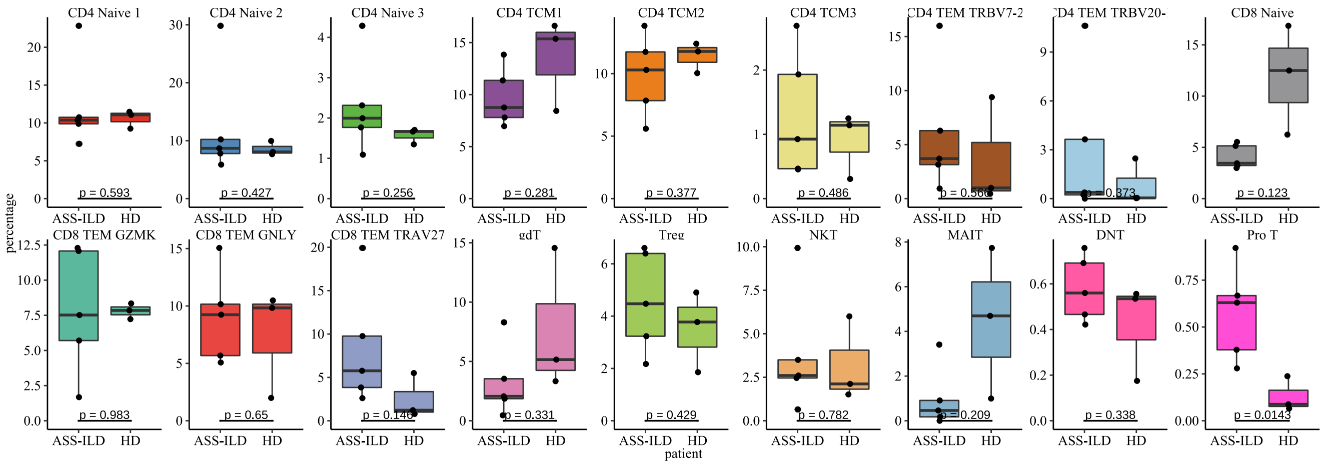

H

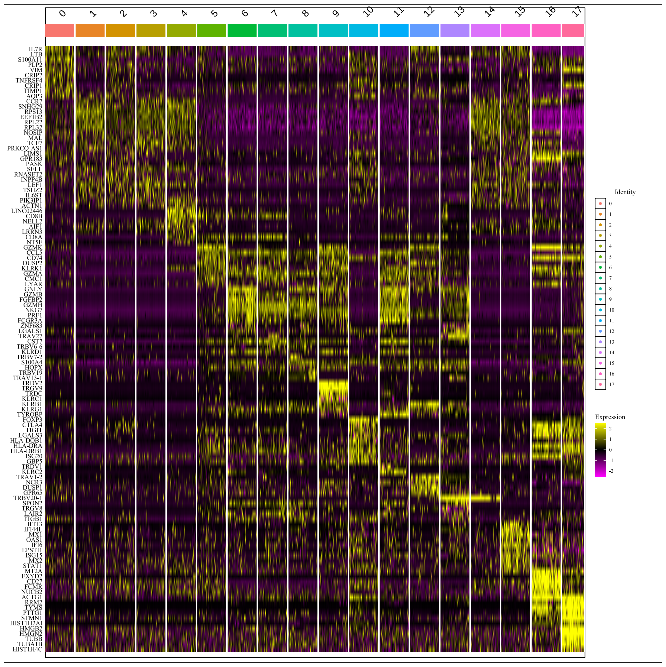

Supplementary Figure 4 Immunological features of T cell subsets before integration. **(A)** UMAP visualization of 26843 T cells, including 9 CD4+ T cell subsets, 4 CD8+ T cell subsets, and 5 other T cell subsets. **(B)** Annotating condition of each patient with ASS-ILD and HD. **(C)** Expressions of canonical marker genes for 18 T cell subsets as represented in the UMAP plot. **(D)** Violin plot depicting the expression distribution of selected marker genes in the 18 T cell subsets. The rows represent selected marker genes and the columns represent clusters. **(E)** Dot plots depicting average expression and percentage of expressed cells of selected marker genes in each labeled T cell subset. **(F)** Bar plot depicting cell compositions of each sample. **(G)** Bar plot depicting average cell percentage of each cluster between patients with ASS-ILD and HDs. Error bars represent mean ± s.e.m. for 5 patients and 3 HDs. **(H)** Heatmap showing the up- and down-regulated genes in T subsets. TCM, central memory T lymphocyte; TEM, effector memory T lymphocyte; Treg, regulatory CD4 T lymphocyte;

NKT, natural killer T cell; MAIT, mucosal-associated invariant T cell; DNT, double negative T cell; pro T, proliferative T cell; UMAP, uniform manifold approximation and projection; ASS-ILD, anti-synthetase syndrome-associated interstitial lung disease; HD, healthy donor; s.e.m., standard error of mean.

A

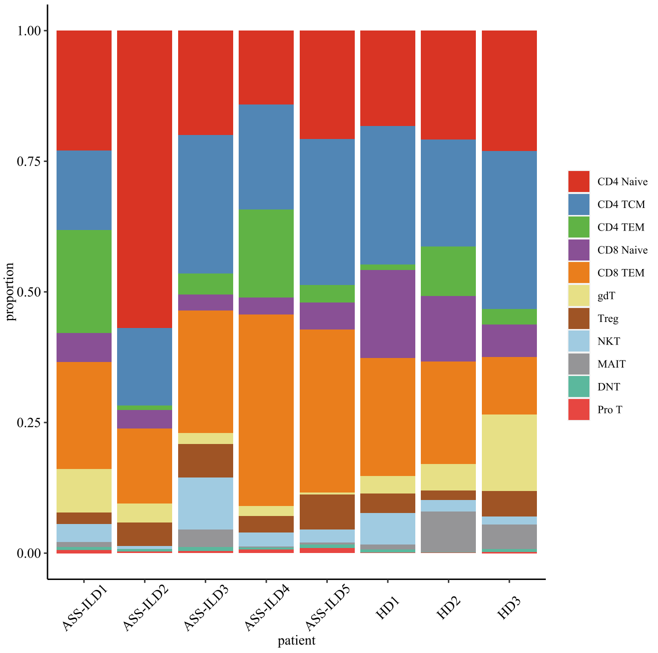

B

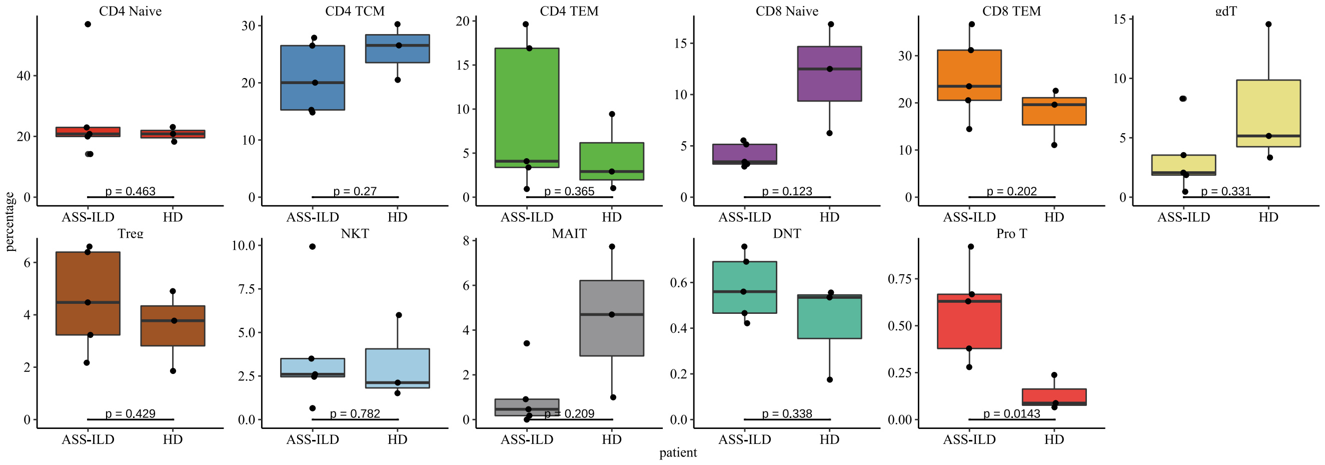

C

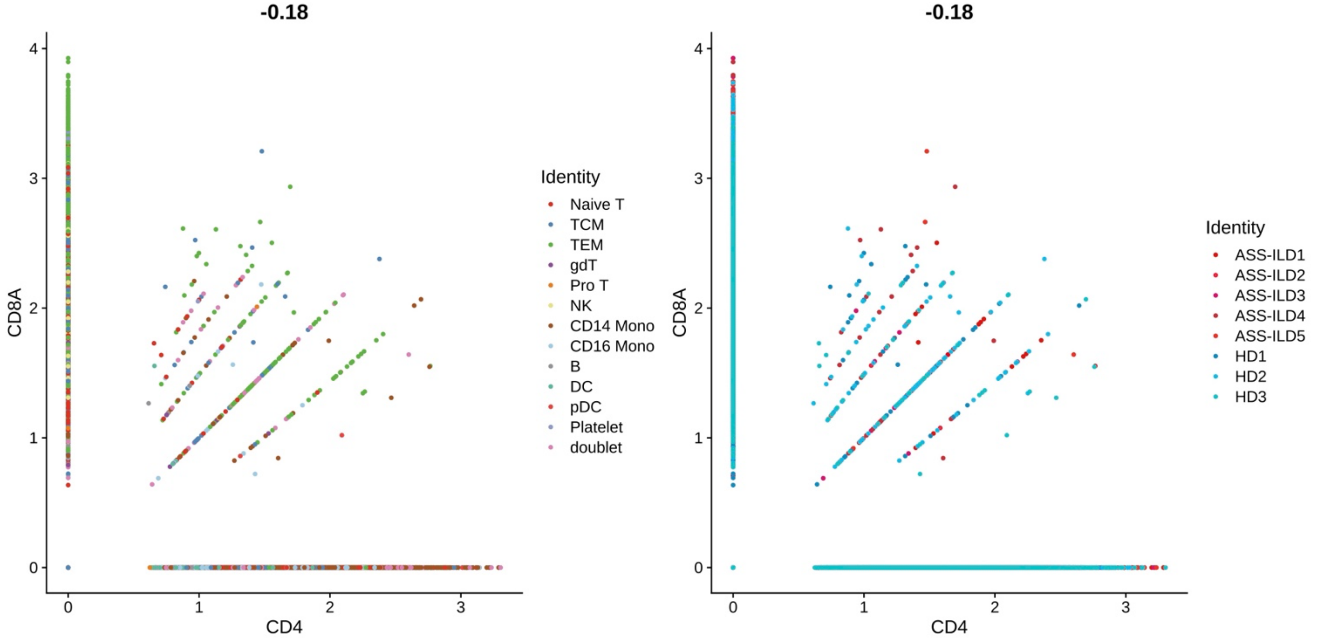

Supplementary Figure 5 Bar plots showing cell proportions of T subsets after integration, and expression distribution of CD4 and CD8A. (A) Bar plot depicting cell proportion of each sample. (**B**) Bar plot depicting average cell percentage of each cluster between patients with ASS-ILD and HDs. Error bars represent mean ± s.e.m. for 5 patients and 3 HDs. **(C)** Expression distribution of CD4 and CD8 in each cell type (left) and individual (right). ASS-ILD, anti-synthetase syndrome-associated interstitial lung disease; HD, healthy donors; TCM, central memory T lymphocyte; TEM, effector memory T lymphocytes; Treg, regulatory CD4 T lymphocyte; NKT, natural killer T cell; MAIT, mucosal-associated invariant T cell; DNT, double negative T cell; pro T, proliferative T cell; mono, monocyte; DC, dendritic cell; pDC, plasmacytoid DC; s.e.m., standard error of mean.

A

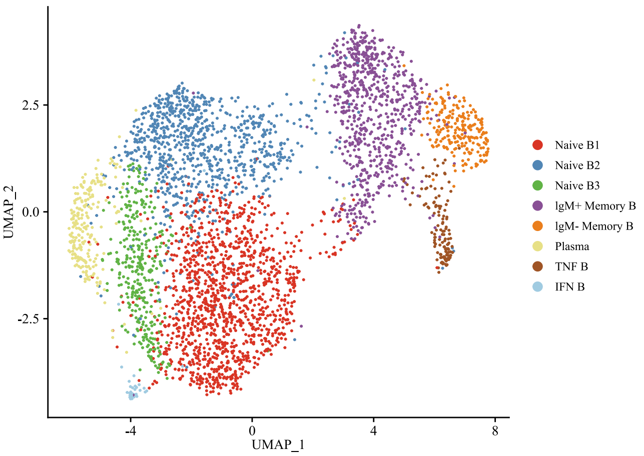

B

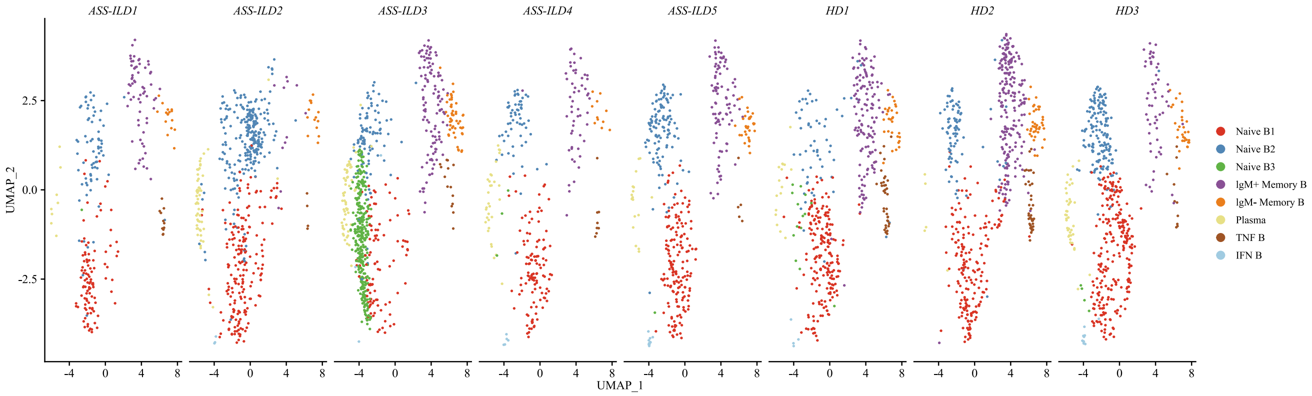
ASS-ILD1
ASS-ILD2
ASS-ILD3
ASS-ILD4
ASS-ILD5
HD1
HD2
HD3
UMAP_2
UMAP_1
Naive B1
Naive B2
Naive B3
IgM+ Memory B
IgM- Memory B
Plasma
TNF B
IFN B

C

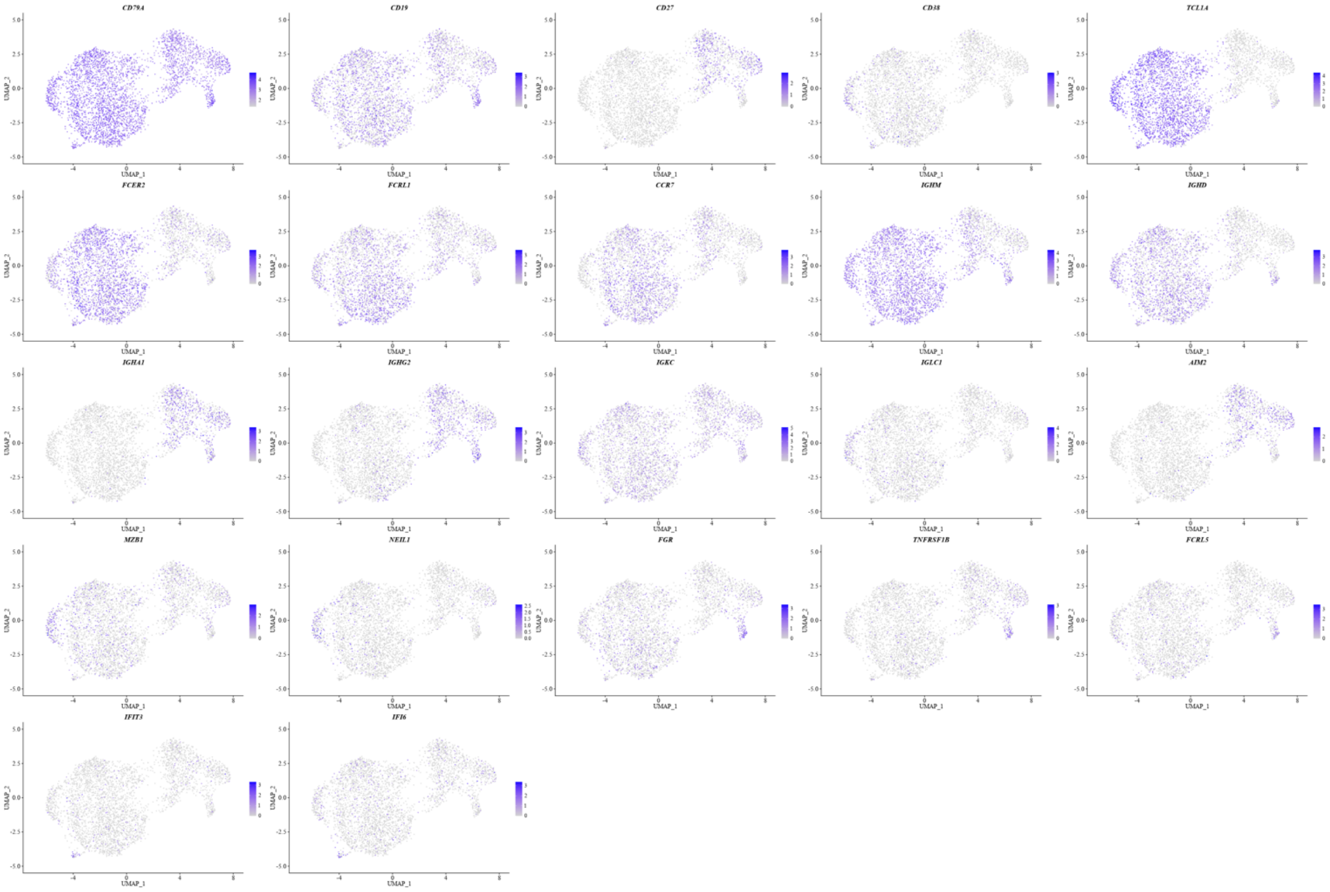
CD79A
CD19
CD27
CD38
TCL1A
FCER2
FCRL1
CCR7
IGHM
IGHD
IGHA1
IGHG2
IGKC
IGLC1
AIM2
MZB1
NEIL1
FGR
TNFRSF1B
FCRL5
IFIT3
IFI6

D

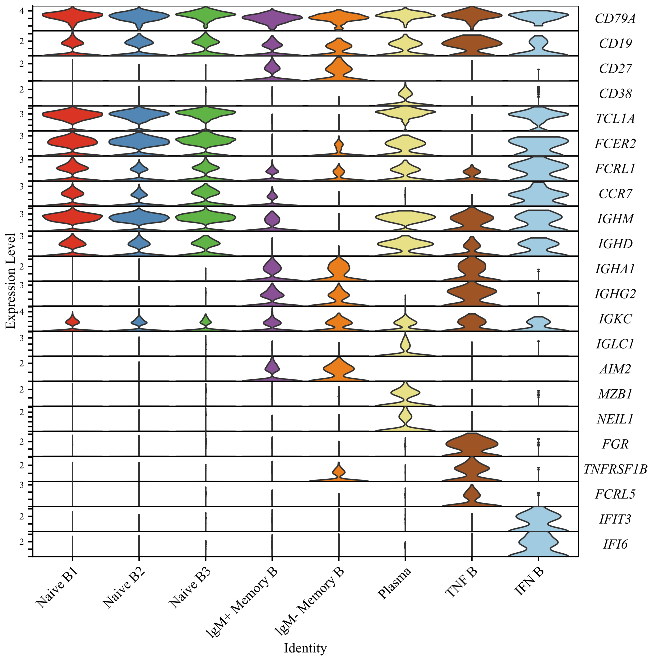
CD79A
CD19
CD27
CD38
TCL1A
FCER2
FCRL1
CCR7
IGHM
IGHD
IGHA1
IGHG2
IGKC
IGLC1
AIM2
MZB1
NEIL1
FGR
TNFRSF1B
FCRL5
IFIT3
IFI6
Expression Level
Naive B1
Naive B2
Naive B3
IgM+ Memory B
IgM- Memory B
Plasma
TNF B
IFN B
Identity

E

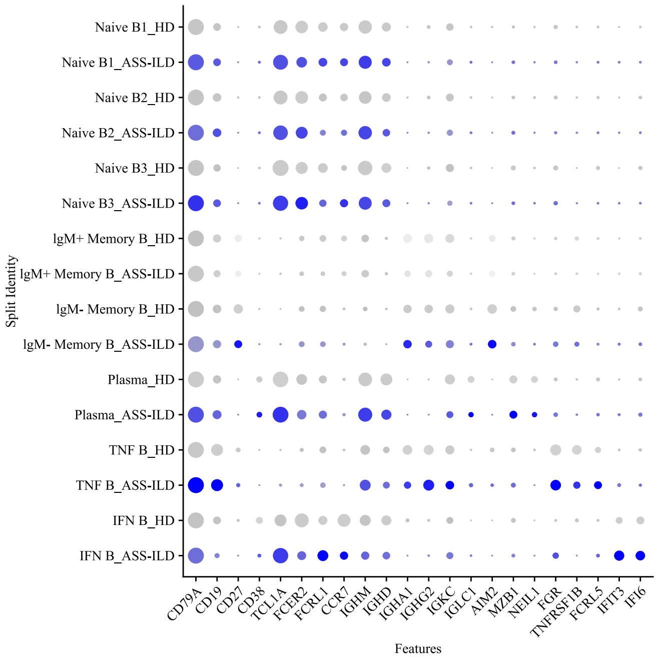
Naive B1_HD
Naive B1_ASS-ILD
Naive B2_HD
Naive B2_ASS-ILD
Naive B3_HD
Naive B3_ASS-ILD
IgM+ Memory B_HD
IgM+ Memory B_ASS-ILD
IgM- Memory B_HD
IgM- Memory B_ASS-ILD
Plasma_HD
Plasma_ASS-ILD
TNF B_HD
TNF B_ASS-ILD
IFN B_HD
IFN B_ASS-ILD
Split Identity
CD79A
CD19
CD27
CD38
TCL1A
FCER2
FCRL1
CCR7
IGHM
IGHD
IGHA1
IGHG2
IGKC
IGLC1
AIM2
MZB1
NEIL1
FGR
TNFRSF1B
FCRL5
IFIT3
IFI6
Features

F

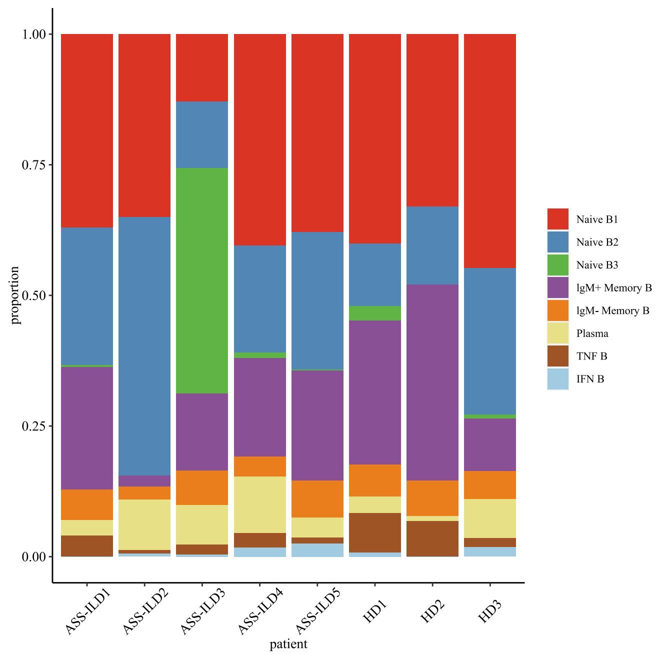
1.00
0.75
0.50
0.25
0.00
proportion
Naive B1
Naive B2
Naive B3
IgM+ Memory B
IgM- Memory B
Plasma
TNF B
IFN B
ASS-ILD1
ASS-ILD2
ASS-ILD3
ASS-ILD4
ASS-ILD5
HD1
HD2
HD3
patient

G

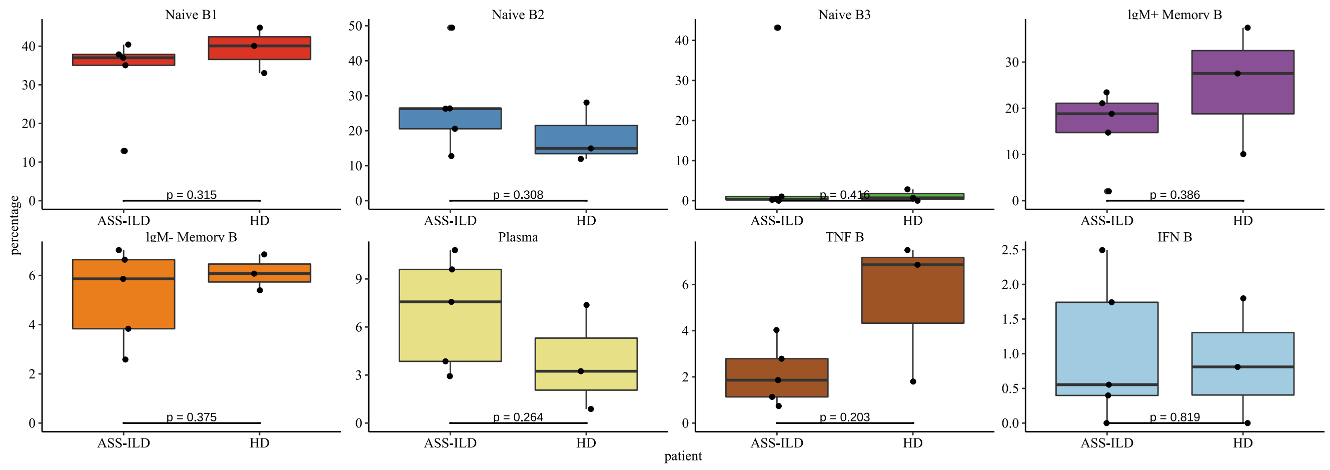
percentage
Naive B1
p = 0.315
Naive B2
p = 0.308
Naive B3
p = 0.416
IgM+ Memory B
p = 0.386
IgM- Memory B
p = 0.375
Plasma
p = 0.264
TNF B
p = 0.203
IFN B
p = 0.819
ASS-ILD
HD
patient

H

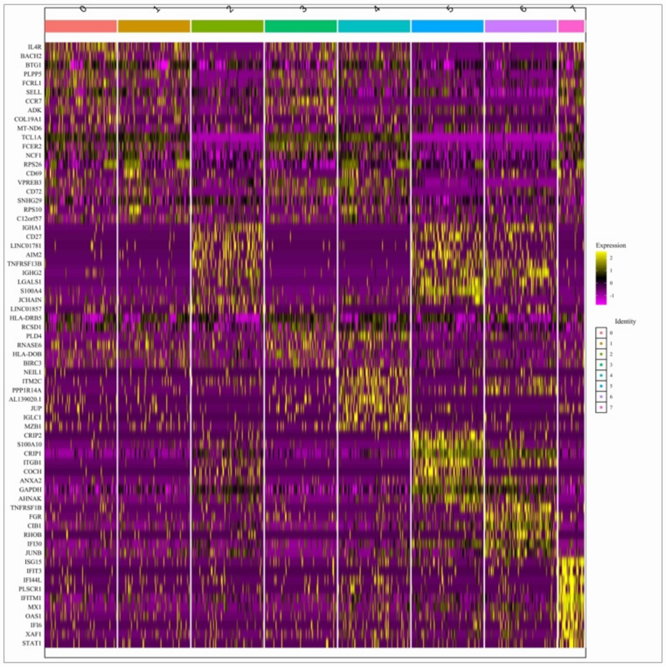

Supplementary Figure 6 Immunological features of B cell subsets before integration. **(A)** UMAP visualization of 4709 B cells, including three Naïve B cell subsets, two memory B cell subsets, a plasma subset, a TNF B subset, and a IFN B cell subset. **(B)** Annotating condition of each patient with ASS-ILD and HD. **(C)** Expression of canonical marker genes for 8 B cell subsets as represented in the UMAP plot. **(D)** Violin plot depicting the expression distribution of selected marker genes in the 8 B cell subsets. The rows represent selected marker genes and the columns represent clusters. **(E)** Dot plot depicting average expression and percentage of expressed cells of selected marker genes in each labeled B cell subset. **(F)** Bar plot depicting cell compositions of each sample. **(G)** Bar plot depicting average cell percentage of each cluster between patients with ASS-ILD and HDs. Error bars represent mean ± s.e.m. for 5 patients and 3 HDs. **(H)** Heatmap showing the up- and down-regulated genes in B subsets. UMAP, uniform manifold approximation and projection; ASS-ILD, anti-synthetase syndrome-associated interstitial lung disease; HD, healthy donors; s.e.m., standard error of mean.

A

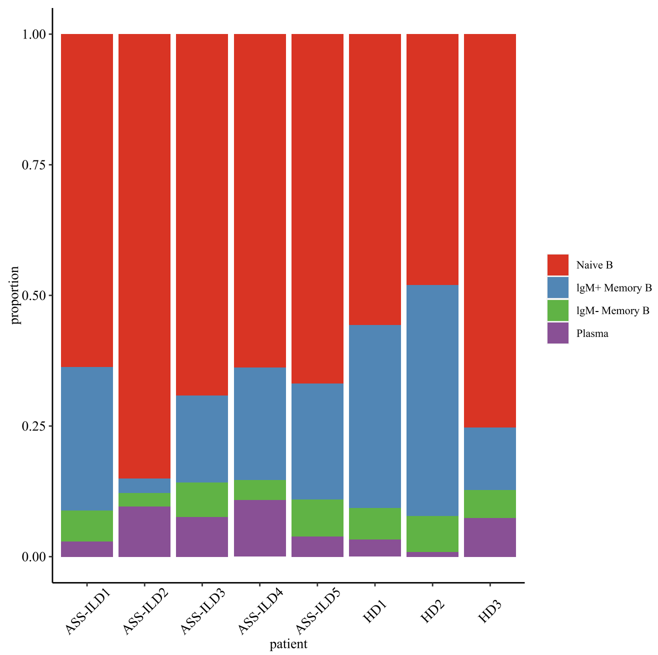

B

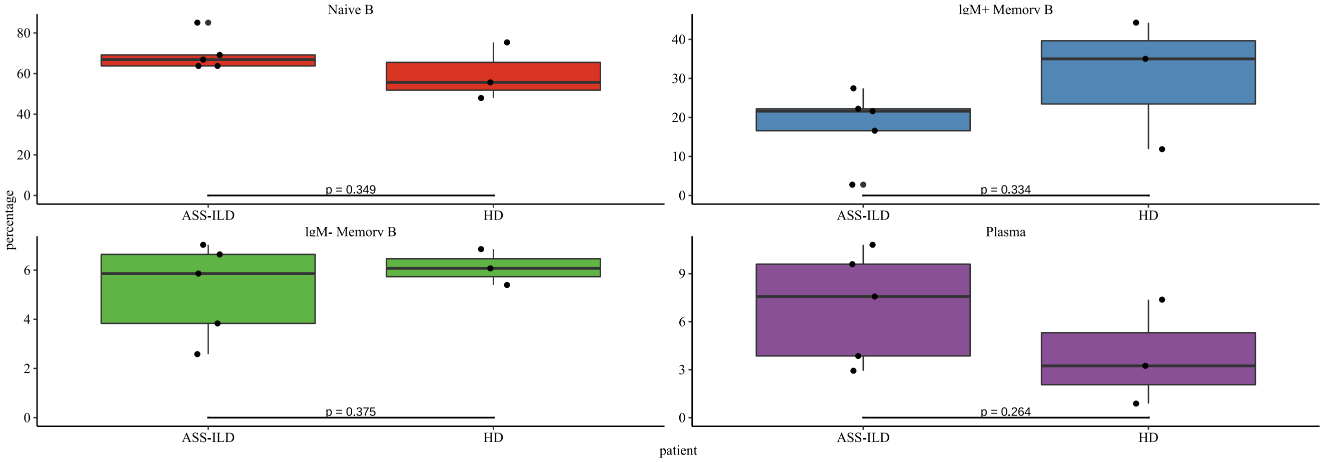

Supplementary Figure 7 Bar plots showing cell proportions of B subsets after integration. (A) Bar plot depicting cell compositions of each sample. (**B**) Bar plot depicting average cell percentage of each cluster between patients with ASS-ILD and HDs. Error bars represent mean ± s.e.m. for 5 patients and 3 HDs. ASS-ILD, anti-synthetase syndrome-associated interstitial lung disease; HD, healthy donors; s.e.m., standard error of mean.
